# An atlas of genetic determinants of forearm fracture

**DOI:** 10.1038/s41588-023-01527-3

**Published:** 2023-11-02

**Authors:** Maria Nethander, Sofia Movérare-Skrtic, Anders Kämpe, Eivind Coward, Ene Reimann, Louise Grahnemo, Éva Borbély, Zsuzsanna Helyes, Thomas Funck-Brentano, Martine Cohen-Solal, Juha Tuukkanen, Antti Koskela, Jianyao Wu, Lei Li, Tianyuan Lu, Maiken E. Gabrielsen, Estonian Biobank Research Team, Reedik Mägi, Mari Hoff, Ulf H. Lerner, Petra Henning, Henrik Ullum, Christian Erikstrup, Søren Brunak, DBDS Genomic Consortium, Arnulf Langhammer, Tiinamaija Tuomi, Asmundur Oddsson, Kari Stefansson, Ulrika Pettersson-Kymmer, Sisse Rye Ostrowski, Ole Birger Vesterager Pedersen, Unnur Styrkarsdottir, Outi Mäkitie, Kristian Hveem, J. Brent Richards, Claes Ohlsson

**Affiliations:** 1 Department of Internal Medicine and Clinical Nutrition, Institute of Medicine, Sahlgrenska Osteoporosis Centre, Centre for Bone and Arthritis Research at the Sahlgrenska Academy, University of Gothenburg, Gothenburg, Sweden; 2 Bioinformatics Core Facility, Sahlgrenska Academy, University of Gothenburg, Gothenburg, Sweden; 3 Department of Molecular Medicine and Surgery, Karolinska Institutet, Stockholm, Sweden; 4 Institute for Molecular Medicine Finland (FIMM), University of Helsinki, Helsinki, Finland; 5 K.G. Jebsen Center for Genetic Epidemiology, Department of Public Health and Nursing, NTNU, Norwegian University of Science and Technology, Trondheim, Norway; 6 Estonian Genome Center, Institute of Genomics, University of Tartu, Tartu, Estonia; 7 Department of Pharmacology and Pharmacotherapy, Medical School, University of Pécs, Pécs, Hungary; 8 National Laboratory for Drug Research and Development, Budapest, Hungary; 9 Eotvos Lorand Research Network, Chronic Pain Research Group, University of Pécs, Pécs, Hungary; 10 BIOSCAR UMRS 1132, Université Paris Diderot, Sorbonne Paris Cité, INSERM, Paris, France; 11 Department of Anatomy and Cell Biology, Faculty of Medicine, Institute of Cancer Research and Translational Medicine, University of Oulu, Oulu, Finland; 12 Lady Davis Institute for Medical Research, Jewish General Hospital, Montreal, Quebec Canada; 13 Department of Neuromedicine and Movement Science, Norwegian University of Science and Technology, Trondheim, Norway; 14 Department of Rheumatology, St Olavs Hospital, Trondheim, Norway; 15 Statens Serum Institut, Copenhagen, Denmark; 16 Department of Clinical Immunology, Aarhus University Hospital, Aarhus, Denmark; 17 Department of Clinical Medicine, Aarhus University, Aarhus, Denmark; 18 Novo Nordisk Foundation Center for Protein Research, Faculty of Health and Medical Sciences, University of Copenhagen, Copenhagen, Denmark; 19 HUNT Research Centre, Department of Public Health and Nursing, Norwegian University of Science and Technology, and Levanger Hospital, Nord-Trøndelag Hospital Trust, Levanger, Norway; 20 Folkhälsan Research Center, Helsinki, Finland; 21 Lund University Diabetes Centre, Department of Clinical Sciences, Lund University, Malmö, Sweden; 22 Department of Endocrinology, Abdominal Center, Helsinki University Hospital, Helsinki, Finland; 23 Research Program for Clinical and Molecular Metabolism, Faculty of Medicine, University of Helsinki, Helsinki, Finland; 24 deCODE genetics, Reykjavik, Iceland; 25 Faculty of Medicine, University of Iceland, Reykjavik, Iceland; 26 Department of Integrative Medical Biology, Clinical Pharmacology, Umea University, Umea, Sweden; 27 Department of Clinical Medicine, Faculty of Health and Medical Sciences, University of Copenhagen, Copenhagen, Denmark; 28 Department of Clinical Immunology, Copenhagen Hospital Biobank Unit, Copenhagen University Hospital Rigshospitalet, Copenhagen, Denmark; 29 Department of Clinical Medicine, Faculty of Health and Medical Science, University of Copenhagen, Copenhagen, Denmark; 30 Department of Clinical Immunology, Zealand University Hospital, Koege, Denmark; 31 Folkhälsan Institute of Genetics, Helsinki, Finland; 32 Children’s Hospital and Pediatric Research Center, University of Helsinki and Helsinki University Hospital, Helsinki, Finland; 33 Department of Human Genetics, McGill University, Montreal, Quebec Canada; 34 Department of Twin Research and Genetic Epidemiology, King’s College London, London, UK; 35 Region Västra Götaland, Sahlgrenska University Hospital, Department of Drug Treatment, Gothenburg, Sweden

**Keywords:** Genetics research, Translational research

## Abstract

Osteoporotic fracture is among the most common and costly of diseases. While reasonably heritable, its genetic determinants have remained elusive. Forearm fractures are the most common clinically recognized osteoporotic fractures with a relatively high heritability. To establish an atlas of the genetic determinants of forearm fractures, we performed genome-wide association analyses including 100,026 forearm fracture cases. We identified 43 loci, including 26 new fracture loci. Although most fracture loci associated with bone mineral density, we also identified loci that primarily regulate bone quality parameters. Functional studies of one such locus, at *TAC4*, revealed that *Tac4*^–*/*–^ mice have reduced mechanical bone strength. The strongest forearm fracture signal, at *WNT16*, displayed remarkable bone-site-specificity with no association with hip fractures. Tall stature and low body mass index were identified as new causal risk factors for fractures. The insights from this atlas may improve fracture prediction and enable therapeutic development to prevent fractures.

## Abstract (web summary)

Genome-wide analyses identify 43 loci associated with forearm fracture, including some influencing bone quality parameters. Follow-up work shows that *Tac4* knockout mice exhibit reduced mechanical bone strength with no effect on bone mineral density.

## Main

Osteoporosis is a common skeletal disease, leading to a reduction in bone density and quality, and increased fracture risk. One in two elderly women and one in four elderly men will at some point suffer an osteoporotic fracture^1,2^. Osteoporosis treatments used currently increase bone mineral density (BMD) and thereby reduce fracture risk^3^. Besides BMD, bone quality parameters such as bone dimensions, bone microstructure and bone matrix composition may contribute to fracture risk; identification of BMD-independent bone quality mechanisms for fractures may thus point toward potential novel drug targets, which could potentially work synergistically with BMD-increasing medicines.

There is a considerable genetic contribution to osteoporotic fractures, with the magnitude of fracture heritability differing between fractures at different bone sites. Twin studies have shown a heritability estimate of ~50% for the two main nonvertebral fractures—hip and forearm fractures—but it is lower (24%) for vertebral fractures^4–6^. The heritable component of fracture risk is proposed to be partly independent of BMD^4,7^. It is therefore likely that part of the heritable predisposition is mediated by genetic influences on bone quality parameters, not captured by dual-energy X-ray absorptiometry (DXA), or on nonskeletal factors such as neuromuscular control and cognition, which influence the risk of falling.

Most genome-wide association studies (GWAS) performed previously on osteoporosis focused on BMD as the outcome^8–11^. These studies have identified several genetic BMD signals, and previous Mendelian randomization (MR) studies have revealed that low BMD, measured by different areal BMD (aBMD) parameters analyzed by DXA or estimated BMD (eBMD) using ultrasound in the heel, is a strong causal risk factor for fractures at any bone site^8,10–12^ and for hip fractures^13^. Thus far, the largest fracture GWAS on fractures at any bone sites (*n* = 37,857 fracture cases) identified only BMD-dependent fracture signals^12^. It is possible that the mechanism of fracture varies for different bone sites and that the only principal common causal risk factor is low BMD. Thus, bone-site-specific BMD-independent fracture signals might exist, but the strength of these will be diluted in combined GWAS of fractures at different bone sites. This is important because, if the causal proteins for fractures differ partly by fracture site, then medicines should be developed also focusing on these specific sites. We hypothesize that the likelihood of identifying BMD-independent fracture signals will be improved in a GWAS that includes only one well-defined and bone-site-specific fracture in a well-powered setting. To this end, we selected forearm fractures, as these fractures occur relatively early in life when fracture heritability is high^5^. We propose that the early age of forearm fractures, before acquisition of main age-dependent BMD changes, may enhance the likelihood of identifying non-BMD fracture loci with impact on bone-site-specific cortical bone dimensions, trabecular bone microstructure and other bone quality parameters. We identified as many as 100,026 forearm fracture cases using eight Northern European biobanks, enabling a well-powered forearm fracture GWAS meta-analysis, followed by replication.

## Results

### GWAS and meta-analyses

Using a discovery set of 50,471 forearm fracture cases and 970,623 controls (Supplementary Tables 1 and 2), we identified in total 59 independent signals at genome-wide significant (GWS) level (*P* < 5 × 10^−8^; Supplementary Fig. 1a,b). Of these, 50 association signals from 43 loci replicated (*P* < 0.05 with the effect estimate in the same direction as in the discovery analysis) when evaluated in the replication cohorts (Supplementary Tables 3 and 4) comprising 49,555 fracture cases and 620,360 controls (Table 1 and Supplementary Table 5).

**Table 1:** Conditionally independent GWS variants from the main model

							Discovery *n* cases = 50,471 *n* controls = 969,623			Replication *n* cases = 49,555 *n* controls = 620,360			Combined *n* cases = 100,026 *n* controls = 1,589,983							
Locus no.	SNP	Position	Closest gene	EA	OA	EAF	OR	95% CI	*P*	OR	95% CI	*P*	OR	95% CI	*P*	New fracture locus	New bone locus	Locus GWS associated with eBMD	Locus GWS associated with aBMD	Coding effect
1	rs9287237	chr1:240597214	*FMN2/GREM2*	G	T	0.85	1.06	(1.04–1.08)	8.3 × 10^−^^9^	1.06	(1.04–1.08)	9.5 × 10^−^^10^	1.06	(1.05–1.08)	4.4 × 10^−^^17^	Yes		Yes	Yes	Intronic
2	rs6726855	chr2:40636180	*SLC8A1*	T	G	0.27	1.06	(1.04–1.08)	1.6 × 10^−12^	1.06	(1.04–1.08)	3.2 × 10^−^^9^	1.06	(1.05–1.07)	3.1 × 10^−^^20^			Yes	Yes	Intronic
3	rs687914	chr2:45878760	*PRKCE*	T	G	0.22	1.06	(1.04–1.08)	5.6 × 10^−^^10^	1.05	(1.03–1.07)	1.0 × 10^−^^7^	1.05	(1.04–1.07)	3.7 × 10^−^^16^	Yes		Yes		UTR5
4	rs4671960	chr2:54798541	*SPTBN1*	G	A	0.28	1.05	(1.03–1.07)	2.9 × 10^−^^9^	1.04	(1.02–1.06)	4.6 × 10^−^^6^	1.04	(1.03–1.06)	9.0 × 10^−^^14^			Yes		Intronic
5	rs55983207	chr2:119529829	*EN1*	T	C	0.92	1.16	(1.13–1.20)	4.1 × 10^−^^25^	1.14	(1.10–1.18)	6.2 × 10^−^^14^	1.15	(1.13–1.18)	3.4 × 10^−37^	Yes		Yes	Yes	Intergenic
5	rs144279715	chr2:119548256	*EN1*	A	G	0.98	1.20	(1.14–1.26)	1.8 × 10^−^^13^	1.16	(1.10–1.23)	6.9 × 10^−^^8^	1.18	(1.14–1.23)	9.7 × 10^−^^20^					Intergenic
6	rs10931982	chr2:202832130	*FZD7*	T	C	0.20	1.06	(1.04–1.08)	5.4 × 10^−^^11^	1.04	(1.02–1.06)	3.1 × 10^−^^5^	1.05	(1.04–1.07)	2.6 × 10^−^^14^	Yes		Yes	Yes	Intergenic
7	rs419918	chr3:41118898	*CTNNB1*	G	C	0.45	1.06	(1.04–1.07)	9.2 × 10^−^^15^	1.03	(1.02–1.05)	1.9 × 10^−^^5^	1.05	(1.04–1.06)	9.7 × 10^−^^18^			Yes	Yes	Intergenic
8	rs4505759	chr4:1003022	*FGFRL1*	C	T	0.69	1.06	(1.05–1.08)	1.5 × 10^−^^13^	1.06	(1.04–1.08)	3.3 × 10^−^^12^	1.06	(1.05–1.07)	3.2 × 10^−^^24^			Yes	Yes	Upstream
8	rs78520297	chr4:1004864	*FGFRL1*	C	T	0.15	1.09	(1.07–1.12)	7.2 × 10^−^^17^	1.06	(1.04–1.08)	8.7 × 10^−^^8^	1.08	(1.06–1.09)	3.4 × 10^−^^22^					UTR5
8	rs113061374	chr4:1052662	*FGFRL1*	T	C	0.04	1.13	(1.09–1.18)	6.8 × 10^−^^11^	1.06	(1.02–1.10)	9.0 × 10^−^^4^	1.09	(1.07–1.12)	5.6 × 10^−12^					Intronic
9	rs4395467	chr4:88645344	*DMP1*	C	T	0.47	1.05	(1.03–1.06)	1.1 × 10^−^^10^	1.02	(1.00–1.03)	1.4 × 10^−^^2^	1.03	(1.02–1.04)	1.5 × 10^−^^10^	Yes		Yes		ncRNA_intronic
10	rs3957282	chr6:44867836	*SUPT3H*	T	C	0.05	1.10	(1.07–1.14)	2.9 × 10^−^^9^	1.05	(1.02–1.09)	2.5 × 10^−^^3^	1.08	(1.05–1.11)	1.6 × 10^−^^10^	Yes		Yes		Intronic
11	rs915125	chr6:82463376	*TENT5A*	C	T	0.71	1.05	(1.03–1.06)	1.0 × 10^−^^9^	1.03	(1.01–1.05)	2.6 × 10^−^^4^	1.04	(1.03–1.05)	3.6 × 10^−^^12^	Yes	Yes			Upstream
12	rs577721086	chr6:127440047	*RSPO3*	T	C	0.94	1.11	(1.08–1.15)	9.0 × 10^−^^10^	1.10	(1.07–1.14)	2.4 × 10^−^^10^	1.11	(1.08–1.13)	1.4 × 10^−^^18^			Yes	Yes	UTR5
12	rs9482773	chr6:127459552	*RSPO3*	G	C	0.52	1.11	(1.10–1.13)	1.7 × 10^−^^50^	1.08	(1.06–1.10)	3.0 × 10^−^^24^	1.10	(1.09–1.11)	3.8 × 10^−^^71^					Intronic
13	rs1891002	chr6:151900047	*CCDC170*	A	T	0.28	1.08	(1.07–1.10)	3.6 × 10^−^^24^	1.05	(1.03–1.06)	1.2 × 10^−^^7^	1.07	(1.05–1.08)	3.3 × 10^−^^28^			Yes	Yes	Intronic
13	rs2941741	chr6:152008982	*ESR1*	G	A	0.54	1.05	(1.03–1.06)	5.0 × 10^−^^10^	1.04	(1.02–1.05)	9.8 × 10^−^^7^	1.04	(1.03–1.05)	3.1 × 10^−^^15^					Intergenic
14	rs28402081	chr7:633179	*PRKAR1B*	T	G	0.19	1.05	(1.03–1.07)	2.1 × 10^−^^8^	1.03	(1.01–1.05)	2.8 × 10^−^^3^	1.04	(1.03–1.06)	7.1 × 10^−^^10^	Yes	Yes			Intronic
15	rs28362709	chr7:30955539	*AQP1*	T	G	0.19	1.08	(1.06–1.10)	7.8 × 10^−^^16^	1.08	(1.07–1.10)	6.4 × 10^−^^19^	1.08	(1.07–1.09)	4.4 × 10^−^^33^			Yes	Yes	Intronic
16	rs6973667	chr7:38152863	*STARD3NL*	A	G	0.66	1.06	(1.04–1.07)	2.1 × 10^−^^14^	1.04	(1.02–1.05)	1.7 × 10^−^^5^	1.05	(1.04–1.06)	1.6 × 10^−^^17^			Yes	Yes	Intergenic
17	rs4389863	chr7:96145105	*C7orf76*	A	G	0.32	1.05	(1.03–1.07)	5.0 × 10^−^^10^	1.05	(1.04–1.07)	4.2 × 10^−^^11^	1.05	(1.04–1.06)	1.4 × 10^−^^19^			Yes	Yes	Intergenic
18	rs2908007	chr7:120962164	*WNT16*	A	G	0.62	1.22	(1.20–1.24)	1.0 × 10^−^^156^	1.11	(1.09–1.12)	1.5 × 10^−^^36^	1.17	(1.15–1.18)	1.8 × 10^−^^174^			Yes	Yes	Intergenic
19	rs117108011	chr8:119901442	*TNFRSF11B*	A	G	0.98	1.24	(1.17–1.30)	3.8 × 10^−^^15^	1.12	(1.05–1.21)	1.3 × 10^−^^3^	1.19	(1.15–1.25)	2.1 × 10^−^^16^	Yes		Yes		Intergenic
20	rs1159798	chr10:54412493	*MBL2*	C	A	0.77	1.06	(1.04–1.08)	1.9 × 10^−^^12^	1.06	(1.04–1.08)	2.8 × 10^−^^10^	1.06	(1.05–1.08)	3.3 × 10^−^^21^			Yes	Yes	ncRNA_intronic
20	rs10824766	chr10:54428297	*MBL2*	T	C	0.12	1.10	(1.07–1.12)	1.6 × 10^−^^16^	1.13	(1.10–1.15)	1.0 × 10^−^^24^	1.11	(1.09–1.13)	7.1 × 10^−^^39^					ncRNA_intronic
21	rs55730604	chr11:16530509	*SOX6*	T	C	0.68	1.05	(1.03–1.06)	4.4 × 10^−^^9^	1.04	(1.02–1.06)	2.3 × 10^−^^6^	1.04	(1.03–1.06)	5.3 × 10^−^^14^	Yes		Yes	Yes	Intronic
22	rs1055447	chr11:47186424	*ARFGAP2*	A	C	0.50	1.05	(1.04–1.07)	1.5 × 10^−^^13^	1.02	(1.00–1.03)	1.8 × 10^−^^2^	1.04	(1.03–1.05)	2.5 × 10^−^^12^	Yes		Yes		UTR3
23	rs4988321	chr11:68174189	*LRP5*	A	G	0.04	1.15	(1.10–1.19)	2.1 × 10^−^^12^	1.10	(1.06–1.14)	7.7 × 10^−^^7^	1.12	(1.09–1.15)	2.8 × 10^−^^17^			Yes	Yes	Nonsynonymous SNV
24	rs755760	chr11:68827175	*TPCN2*	T	A	0.68	1.05	(1.03–1.07)	2.0 × 10^−^^10^	1.02	(1.01–1.04)	8.5 × 10^−^^3^	1.04	(1.03–1.05)	1.4 × 10^−10^	Yes		Yes		Intronic
25	rs477944	chr11:86860509	*TMEM135*	A	G	0.31	1.10	(1.08–1.11)	3.7 × 10^−^^32^	1.05	(1.03–1.06)	4.0 × 10^−^^8^	1.07	(1.06–1.08)	3.7 × 10^−^^35^			Yes	Yes	Intronic
26	rs10749999	chr11:112394430	*PLET1*	G	A	0.63	1.04	(1.03–1.06)	4.1 × 10^−^^8^	1.04	(1.02–1.05)	1.6 × 10^−^^6^	1.04	(1.03–1.05)	3.3 × 10^−^^13^	Yes		Yes		ncRNA_intronic
27	rs215226	chr12:591300	*B4GALNT3*	A	G	0.62	1.04	(1.03–1.06)	1.3 × 10^−^^8^	1.04	(1.02–1.05)	1.9 × 10^−^^6^	1.04	(1.03–1.05)	1.3 × 10^−^^13^	Yes		Yes		Intronic
28	rs2926799	chr12:49263634	*RND1*	A	G	0.81	1.06	(1.04–1.08)	2.3 × 10^−^^9^	1.04	(1.02–1.06)	1.3 × 10^−^^4^	1.05	(1.03–1.06)	3.7 × 10^−^^12^	Yes		Yes		Intergenic
29	rs5800113	chr12:94142186	*CRADD*	A	AT	0.55	1.04	(1.03–1.06)	1.9 × 10^−^^8^	1.02	(1.01–1.04)	3.6 × 10^−^^3^	1.03	(1.02–1.04)	1.5 × 10^−^^9^	Yes		Yes		Intronic
30	rs11840862	chr13:42956463	*AKAP11*	G	A	0.71	1.04	(1.03–1.06)	4.7 × 10^−^^8^	1.03	(1.01–1.05)	6.8 × 10^−^^4^	1.04	(1.03–1.05)	2.5 × 10^−^^10^	Yes		Yes	Yes	Intergenic
31	rs4444235	chr14:54410919	*BMP4*	T	C	0.56	1.05	(1.03–1.06)	1.1 × 10^−^^10^	1.03	(1.02–1.05)	6.1 × 10^−^^5^	1.04	(1.03–1.05)	8.8 × 10^−^^14^	Yes		Yes		Intergenic
32	rs1467561	chr14:103994961	*TRMT61A*	C	T	0.34	1.06	(1.04–1.07)	1.6 × 10^−^^13^	1.03	(1.02–1.05)	7.5 × 10^−^^5^	1.05	(1.03–1.06)	6.1 × 10^−^^16^	Yes		Yes		ncRNA_exonic
33	rs11632429	chr15:38341268	*TMCO5A*	G	C	0.85	1.06	(1.04–1.08)	5.5 × 10^−^^9^	1.03	(1.01–1.05)	8.0 × 10^−^^3^	1.05	(1.03–1.06)	8.5 × 10^−^^10^	Yes			Yes	ncRNA_intronic
34	rs7169993	chr15:70506192	*TLE3*	G	A	0.34	1.04	(1.03–1.06)	1.0 × 10^−^^8^	1.02	(1.00–1.04)	1.2 × 10^−^^2^	1.03	(1.02–1.04)	3.8 × 10^−^^9^	Yes		Yes		Intergenic
35	rs71378512	chr16:410178	*AXIN1*	A	G	0.06	1.10	(1.06–1.13)	5.7 × 10^−^^9^	1.05	(1.02–1.09)	2.0 × 10^−^^3^	1.08	(1.05–1.10)	2.5 × 10^−^^10^	Yes		Yes	Yes	Intergenic
36	rs62028332	chr16:51025468	*SALL1*	G	A	0.87	1.08	(1.06–1.10)	3.9 × 10^−^^12^	1.04	(1.02–1.07)	1.1 × 10^−^^4^	1.06	(1.05–1.08)	2.1 × 10^−^^14^			Yes		Intergenic
37	rs60791151	chr16:55068167	*IRX5*	T	G	0.54	1.05	(1.04–1.06)	6.2 × 10^−^^12^	1.03	(1.01–1.04)	2.4 × 10^−^^4^	1.04	(1.03–1.05)	4.6 × 10^−^^14^	Yes		Yes		Intergenic
38	rs4790881	chr17:2068932	*SMG6*	C	A	0.31	1.05	(1.03–1.06)	1.5 × 10^−^^9^	1.04	(1.02–1.05)	2.1 × 10^−^^5^	1.04	(1.03–1.05)	2.3 × 10^−^^13^	Yes		Yes	Yes	Intronic
39	rs4792909	chr17:41798824	*SOST*	G	T	0.57	1.07	(1.05–1.08)	2.8 × 10^−^^18^	1.06	(1.04–1.08)	3.1 × 10^−^^13^	1.06	(1.05–1.08)	8.2 × 10^−^^30^			Yes	Yes	Intergenic
39	rs80107551	chr17:41808374	*SOST*	C	T	0.90	1.12	(1.09–1.14)	1.9 × 10^−^^17^	1.11	(1.08–1.14)	4.0 × 10^−^^14^	1.11	(1.09–1.13)	5.9 × 10^−^^30^					Intergenic
40	rs79049182	chr17:47928342	*TAC4*	A	T	0.95	1.13	(1.09–1.16)	9.3 × 10^−^^13^	1.09	(1.05–1.13)	1.1 × 10^−^^6^	1.11	(1.08–1.14)	1.0 × 10^−^^17^	Yes	Yes			Intergenic
41	rs4430817	chr18:13682666	*FAM210A*	C	G	0.38	1.08	(1.06–1.09)	2.3 × 10^−^^23^	1.05	(1.03–1.06)	5.0 × 10^−^^9^	1.06	(1.05–1.07)	1.3 × 10^−^^29^			Yes	Yes	Intronic
42	rs77865749	chr18:77216756	*NFATC1*	G	A	0.83	1.06	(1.04–1.08)	1.6 × 10^−^^10^	1.02	(1.00–1.05)	3.0 × 10^−^^2^	1.05	(1.03–1.06)	4.2 × 10^−10^	Yes		Yes		Intronic
43	rs11088458	chr21:40350120	*ETS2*	G	A	0.69	1.06	(1.05–1.08)	2.2 × 10^−^^14^	1.06	(1.04–1.08)	1.7 × 10^−^^10^	1.06	(1.05–1.07)	2.4 × 10^−^^23^			Yes		Upstream

We identified 50 conditionally independent statistically significant signals from 43 different loci associated with risk of forearm fractures and still statistically significant after replication. Results are presented as OR with 95% CI for each added EA. For the discovery part of the meta-analysis, ORs, CIs and *P* values are from the conditional analysis. ORs, CIs and *P* values, not using conditional analysis, are presented in Supplementary Table 5. EA, effect allele; EAF, effect allele frequency; OA, other allele.

Of the identified replicated loci, 26 have not been reported previously as fracture loci (Table 1) and, although most of the identified loci are known aBMD or eBMD loci, three loci (rs915125 at *TENT5A*, also called *FAM46A*; rs28402081 at *PRKAR1B* and rs79049182 at *TAC4*) have not been reported as aBMD or eBMD GWS loci (Table 1 and Supplementary Note 1)^8–11^.

### Bone-site-specificity for forearm fracture signals

We next evaluated whether the identified signals were specific for forearm fractures by comparing their associations with forearm fractures, hip fractures^13^ and fractures at any bone site^10,12^. All the identified GWS top forearm fracture signals except two were also associated with fractures at any bone site (48 single nucleotide polymorphisms (SNPs) passing nominal statistical significance, *P* < 0.05 and 44 SNPs passing conservative Bonferroni-adjusted statistical significance; *P* < 0.001), and 29 of these signals were associated with hip fractures in the same direction as observed for forearm fractures (29 SNPs passing nominal statistical significance (*P* < 0.05), and 17 SNPs passing conservative Bonferroni-adjusted statistical significance (*P* < 0.001); Supplementary Fig. 2)^10,13^. It should be emphasized that the number of cases in the previous GWAS on hip fractures (*n* = 11,516)^13^ was lower compared with the number of fractures in the discovery phase of the present GWAS on forearm fractures (*n* = 50,471)—a difference that may contribute to the limited overlap between the identified forearm fracture signals and hip fractures.

**Fig. 1:**
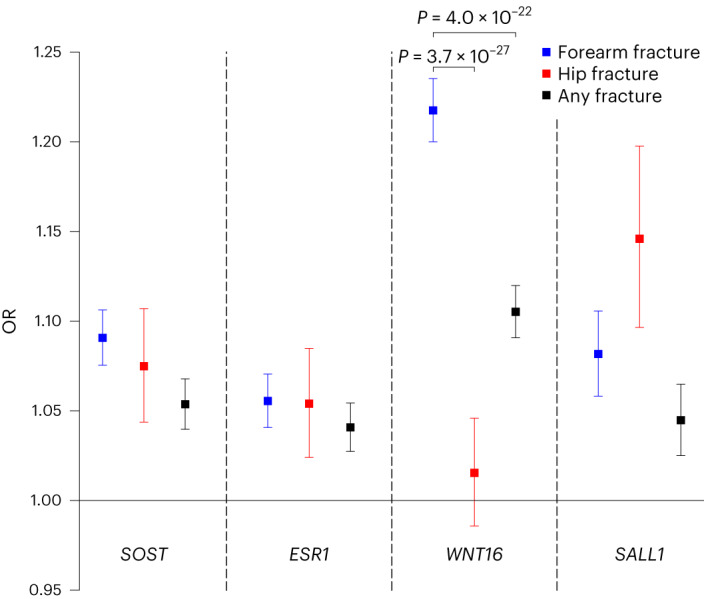
Associations with forearm fracture (50,471 cases and 969,623 controls), hip fracture and any fracture risk for the identified top forearm fracture signal at the *SOST*, *ESR1*, *WNT16* and *SALL1* loci. Data are presented as OR for fracture per effect allele, with 95% CIs (for *SOST*, rs80107551-C; *ESR1*, rs2941741-G; *WNT16*, rs2908007-A; *SALL1*, rs62028332-G). OR for hip fractures are from Nethander et al.^13^ (11,516 cases and 723,838 controls), while OR for any fractures are from Morris et al.^10^ (53,184 cases and 373,611 controls). Statistically significant different associations for forearm fracture compared with the corresponding associations with hip fracture or any fracture are indicated with *P* values. Two-sided *z* test was used to test differences and the statistical significance limit was set to 0.0005 (Bonferroni adjustment considering 50 SNPs and two traits).

The top signal in the most statistically significant forearm fracture locus, *WNT16* (rs2908007), was not associated with hip fractures (*z* test comparing the log odds of the association with hip and forearm fractures *P* = 3.7 × 10^−27^; Fig. 1 and Supplementary Fig. 2), demonstrating that the previously reported association for the *WNT16* signal with fractures at any bone site^10,12^ is driven, to a large extent, by its very strong association with forearm fractures. These findings demonstrate that, although most forearm fracture loci are associated with both forearm fractures and hip fractures, forearm-fracture-specific associations also exist (Fig. 1 and Supplementary Fig. 2). Our observed bone-site-specific associations support extensive clinical observations that antisclerostin treatment^14^ (as indicated by the top *SOST* signal, rs80107551; Fig. 1) and estrogen treatment^15^ (as indicated by the top *ESR1* signal, rs2941741; Fig. 1 and Supplementary Fig. 2) reduce the risk for both hip and forearm fractures, while our present data indicate that potential treatments targeting WNT16 (as indicated by the top *WNT16* signal, rs2908007; Fig. 1) may exert bone-site-specific effects and may not reduce hip fracture risk. In contrast, potential novel treatments targeting, for instance, the *SALL1* signal (as indicated by the top *SALL1*, rs62028332 signal; Fig. 1 and Supplementary Fig. 2) may reduce both hip and forearm fracture risk.

### Functional annotation and expression quantitative trait loci analyses

To search for causal genes associated with risk of forearm fracture, we annotated the 50 identified forearm fracture signals and correlated variants (*r*^2^ > 0.8) with regards to their functional consequences. Signals in two of these loci were predicted to affect coding (missense) of a protein (Supplementary Table 8), including an amino acid substitution in LRP5 (rs4988321, Val667Met). Signals in 26 loci had at least one statistically significant cis expression quantitative trait loci (eQTL) (cis-eQTL) according to GTEx v.8 (false discovery rate <5%; Supplementary Table 9). Furthermore, we observed that the forearm fracture signal at *TAC4* is associated with expression of a *TAC4* antisense transcript (RP11-304F15.3; *P* = 2.6 × 10^−8^, in blood (https://www.eqtlgen.org/). In addition, the forearm fracture signal at *PRKAR1B* is robustly associated with the expression of an antisense transcript to *PRKAR1B* (antisense AC147651.4, *P* = 2.0 × 10^−28^ in blood; GTEx v.8). Finally, using MR, it has been reported that circulating RSPO3 is causally associated with forearm fractures^16^. For further details on functional annotation, eQTLs and pathway analyses, see Supplementary Note 2.

### Genetic determinants of forearm fractures not acting through eBMD

We next aimed to identify loci increasing forearm fracture risk not mediated via decreased eBMD (Supplementary Table 14). To this end, we used multi-trait-based conditional and joint analysis (mtCOJO) and summary statistics from a large eBMD GWAS^10^ to assess genetic influences on forearm fracture risk that are independent of eBMD. We identified genetic signals in nine loci associated with forearm fracture risk after removal of the genetic influence of eBMD (*P* < 5 × 10^−8^; Supplementary Table 15 and Supplementary Fig. 5). The signals in six of these loci (*EN1*, *FGFRL1*, *RSPO3*, *WNT16*, *SOST*, *TAC4*) were also associated with forearm fractures in our main forearm fracture model (Table 1 and Supplementary Table 15).

One locus, *TAC4*, showed effects that seemed not to be mediated through eBMD. *TAC4*, also identified in our main GWAS, resides at a new bone trait locus, and the strength of its association with forearm fracture risk was not affected by removal of the genetic influence of eBMD (Table 1 and Supplementary Table 15). Interestingly, two signals identified in the conditional analyses (rs62621812, *ZNF800*; rs2376600, *ABR*) were not identified in the main forearm fractures GWAS at GWS level, but both these signals were associated with eBMD at GWS level in the opposite direction to what was expected (Supplementary Table 15). Thus, for these two signals, the alleles associated with increased eBMD were associated with increased forearm fracture risk that were nominally statistically significant in the main GWAS and GWS after conditioning on eBMD (Supplementary Table 15). The identified genetic variant at the *ZNF800* locus is a missense SNP (Pro103Ser) in ZNF800, suggesting that *ZNF800* could be the causal gene for this signal.

### *TAC4* regulates bone strength

As described above, we identified three loci (*TENT5A*, *PRKAR1B* and *TAC4*) associated with forearm fractures not reported as GWS in previous fracture or BMD GWAS^8–13^. *TENT5A*, also called *FAM46A*, encodes the TENT5A (terminal nucleotidyltransferase 5A) protein. Although our functional annotation of the top signal, rs915125, at the *TENT5A* locus did not reveal any strong link with the *TENT5A* gene, there are robust mouse and human data, involving disruption of TENT5A function, strongly suggesting that altered function of TENT5A is the underlying mechanism for the genetic signal at the *TENT5A* locus to regulate forearm fracture risk^17,18^. *Tent5a*^–*/*–^ mice have been reported to display affected collagen synthesis and multiple spontaneous fractures, while BMD is mainly unaffected^17^. Furthermore, mutations in *TENT5A* were recently identified in four osteogenesis imperfecta patients with multiple fractures during the first years of life^18^. We consider that the identification of the *TENT5A* locus in our forearm fracture GWAS serves as a positive control, confirming our strategy to identify fracture loci that primarily affect bone quality without any large impact on BMD.

The top signal at the *PRKAR1B* locus, rs28402081, is associated with mRNA expression of *PRKAR1B* (cis-eQTL; Supplementary Table 9). In addition, rs28402081 is strongly associated with the expression of an antisense transcript to *PRKAR1B* (antisense AC147651.4, *P* = 2.0 × 10^−28^ in blood; GTEx v.8). These findings suggest that *PRKAR1B* might be the causal gene for this locus, but further functional studies are required to establish the underlying causal gene. Interestingly, the T allele of rs28402081, associating with increased forearm fracture risk, is nominally associated with increased and not, as expected, decreased forearm BMD (FA-BMD, beta = 0.083 s.d. increase per T allele, *P* = 0.02) (ref. ^11^). To our knowledge, there is no available mouse model with inactivation of the *Prkar1b* gene.

TAC4 (tachykinin precursor 4) was chosen as a strong candidate for further functional studies as (1) the top signal in this locus is associated with the expression of an antisense RNA to *TAC4*, (2) it is a new bone trait locus, (3) the signal is robustly associated with forearm fracture risk in both the discovery and replication cohorts (meta-analysis *P* = 1.0 × 10^−17^) and (4) the strength of its association with forearm fracture risk is not affected by removal of its genetic influence on eBMD (see above). In rodents, there is only one TAC4-related protein, hemokinin 1 (HK1), with a high affinity for the tachykinin NK1 receptor^19^. Previous studies have shown that HK1 competitively inhibits substance P-induced stimulation of osteoclast formation and function in cultured cells and that HK1 immunoreactivity is observed both in osteocytes and osteoclasts in bone^20^. However, the in vivo role of HK1 for bone mass, bone microstructure, bone strength and other possible fracture-related parameters is unknown.

To determine the role of TAC4 and thereby HK1 for bone strength and motor performance in mice, we evaluated 12-month-old *Tac4*^–*/*–^ mice with no remaining *Tac4* mRNA expression (Supplementary Fig. 6a). *Tac4*^*–/*–^ mice were born healthy and had normal tibia length, bodyweight (BW) and height of vertebra L5 (Supplementary Table 16). Previous studies have demonstrated that *Tac4*^*–/–*^ mice display slightly reduced motor performance using the Rotarod test when evaluated 10 days after partial sciatic nerve ligation, but no effect of *Tac4* inactivation on motor performance was observed at baseline before ligation in that study^21^. In the present study, we determined whether the *Tac4*^–*/–*^ mice displayed disturbed motor coordination using the Rotarod test, but no statistically significant difference between groups was observed (Supplementary Fig. 6b). Thus, there does not seem to be any major disturbance in motor performance in 11-month-old *Tac4*^–*/*–^ mice.

**Fig. 2:**
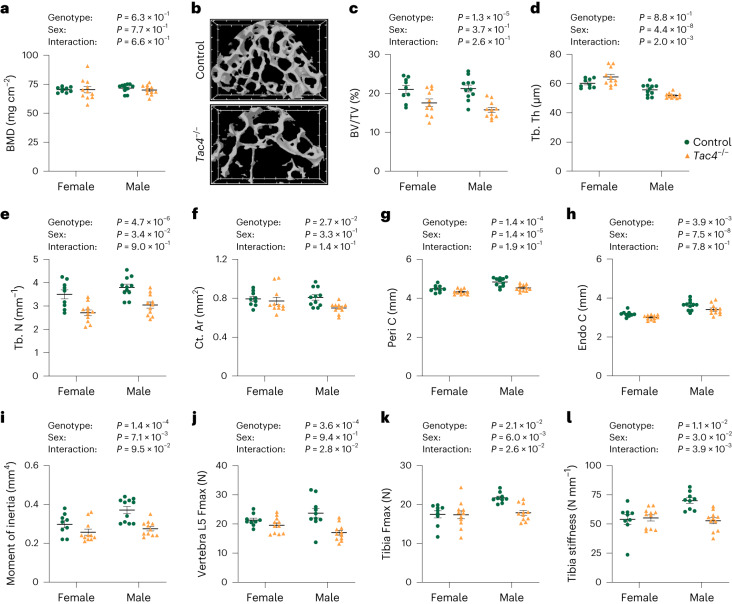
Bone microstructure and bone strength but not aBMD is affected in *Tac4*^–/–^ mice. **a**, Two-dimensional aBMD as measured by DXA in tibia from *Tac4*^–/–^ (female, *n* = 11; male, *n* = 11) mice compared with control (female, *n* = 9; male, *n* = 11) mice. **b**–**e**, µCT measurements of vertebra L5. Representative three-dimensional images of trabecular bone in transversal plane of vertebra L5 from control and *Tac4*^–/–^ male mice (**b**); the distance between tics in the scale grid in *x* and *y* axes in front and back of the three-dimensional image is 200 µm. Trabecular bone volume over total volume (BV/TV) (**c**), trabecular thickness (Tb. Th) (**d**) and trabecular number (Tb. N) (**e**) in vertebra L5 from *Tac4*^–/–^ (female, *n* = 11 male; *n* = 11) mice compared with control (female, *n* = 9; male, *n* = 11) mice. **f**–**i**, Cortical bone area (Ct. Ar) (**f**), periosteal circumference (Peri C) (**g**), endosteal circumference (Endo C) (**h**) and cortical moment of inertia (**i**) in tibia from *Tac4*^–/–^ (female, *n* = 11; male, *n* = 11) mice compared with control (female, *n* = 9; male, *n* = 11) mice. **j**–**l**, Maximal load at failure (Fmax) of vertebra L5 (**j**) as measured by compression test in *Tac4*^–/–^ (female, *n* = 11; male, *n* = 11) mice compared with control (female, *n* = 9; male, *n* = 10) mice. Maximal load at failure (**k**) and stiffness (**l**) of tibia as measured by three-point bending in *Tac4*^–/–^ (female, *n* = 11; male, *n* = 11) mice compared with control (female, *n* = 9; male, *n* = 9) mice. All results refer to 12-month-old mice. Individual values are presented with the mean as horizontal lines and ± s.e.m. as vertical lines. A two-way analysis of variance was used to assess the effects of genotype (*Tac4*^–/–^ or control (*Tac4*^+/+^)), sex (female or male), as well as their interaction. A difference was considered statistically significant when *P* < 0.05.

**Fig. 3:**
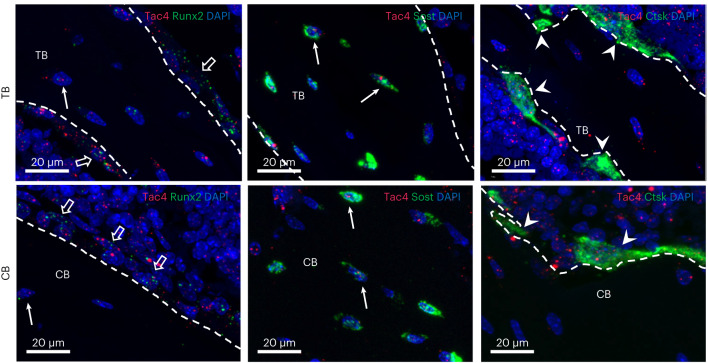
Multiplex FISH on mouse tibiae. Representative maximum intensity projection (×40 magnification) images of mouse tibiae labeled with fluorescence RNAscope for *Tac4* (red) plus *Runx2*, *Sost* or *Ctsk* (green) mRNA and DAPI (blue). *Tac4* and *Runx2* or *Sost* double-positive osteocytes (solid arrows) can be detected both in the trabecular bone (TB) and cortical bone (CB). *Tac4* mRNA also can be found in *Runx2*-expressing osteoblasts (open arrows) and *Ctsk-*expressing osteoclasts (solid arrowheads). Representative maximum intensity projection images are shown of mouse tibiae; *n* = 3 biologically independent mice.

There was also no effect of *Tac4* deficiency on aBMD of the tibia, as analyzed by two-dimensional DXA (Fig. 2a). This finding is in line with the absence of GWS association between the fracture signal at the *TAC4* locus and aBMD as analyzed by DXA in previous human GWAS (refs. ^8,9,11^). However, bone microstructure analyses using CT revealed that *Tac4*^–/–^ mice had substantially reduced trabecular bone volume fraction in the vertebra L5, associated with reduced trabecular number (Fig. 2b–e). In addition, the cortical bone parameters cross-sectional bone area, periosteal circumference and endosteal circumference, as well as the calculated bone strength measure cortical cross-sectional moment of inertia in the diaphyseal region of tibia, were reduced in *Tac4*^–*/*–^ mice (Fig. 2f–i). Mechanical bone strength measurements by compression of vertebra L5 revealed reduced bone strength (maximum load at failure; Fig. 2j) in *Tac4*^–/–^ mice compared with control mice. Cortical bone strength was measured using three-points bending in the diaphyseal region of tibia. The cortical bone strength measures maximal load (Fig. 2k) and bone stiffness (Fig. 2l) were also reduced in *Tac4*^–*/*–^ mice compared with control mice, with slightly more prominent effect in males compared with females (both statistically significant overall genotype effect and statistically significant interaction between genotype and sex in the two-way analysis of variance). Using histomorphometry of vertebral trabecular bone, we observed that the osteoid surface per bone surface (–39.4 ± 10.8%; *P* = 0.013), and mineral apposition rate (–10.6 ± 4.2%; *P* = 0.044) were reduced in *Tac4*^–*/*–^ mice compared with control mice, and there was some evidence of reduced bone formation rate (–25.7 ± 7.9%; *P* = 0.067), while the osteoclast surface per bone surface was not affected (Supplementary Table 17). Collectively, these findings indicate that there is a reduction in bone formation in 12-month-old *Tac4*^*–/*–^ mice, and that this may contribute to the observed reduced bone strength. Fluorescence in situ hybridization (FISH) revealed high expression of *Tac4* mRNA in osteoblast-lineage cells and modest expression in osteoclasts in mice (Fig. 3, Supplementary Fig. 7 and Supplementary Note 3).

### Genetic correlations with risk factors for fractures

We used linkage disequilibrium (LD) score regression (LDSR) to estimate the genetic correlation between forearm fractures and different diseases and traits in humans. We evaluated 18 different genetic correlations for BMD measures and plausible clinical risk factors for fractures (Table 2). FA-BMD was strongly inversely genetically correlated with risk of forearm fractures (*r*_g _= −0.80), and the correlations for eBMD (*r*_g_ = −0.60), femoral neck (FN) BMD (FN-BMD, *r*_g_ = −0.51) and lumbar spine (LS) BMD (LS-BMD, *r*_g_ = −0.44) were moderate (Table 2). Among the other evaluated clinical risk factors, falls and height were positively correlated, whereas body mass index (BMI) was inversely correlated with risk of forearm fracture at Bonferroni-adjusted statistical significance level (*P* < 0.0028, accounting for 18 tests; Table 2). None of the other evaluated risk factors were genetically correlated with forearm fractures (Table 2 and Supplementary Note 4).

**Table 2:** Estimated genetic correlation between fractures and risk factors for fractures

	*r*_g_	*P*
**BMD-related parameters**		
FN-BMD^*^	−0.51	2.9 × 10^−^^18^
LS-BMD*	−0.44	8.7 × 10^−13^
FA-BMD*	−0.80	3.9 × 10^−5^
eBMD*	−0.60	1.1 × 10^−80^
**Clinical risk factors**		
Age at menopause	−0.11	3.8 × 10^−3^
Age at menarche	0.05	1.8 × 10^−1^
Relative age voice broke	0.08	5.6 × 10^−2^
Grip strength	0.03	3.0 × 10^−1^
Vitamin D levels	0.07	5.5 × 10^−^^3^
Falls*	0.24	1.8 × 10^−^^12^
Coronary artery disease	−0.10	5.3 × 10^−^^3^
Rheumatoid arthritis	0.00	9.8 × 10^−^^1^
Inflammatory bowel disease	0.03	5.8 × 10^−^^1^
Type 2 diabetes	−0.09	8.5 × 10^−^^2^
Ever versus never smoked	−0.02	4.5 × 10^−^^1^
Alcohol consumption	0.06	4.2 × 10^−^^2^
Height*	0.09	1.4 × 10^−^^3^
BMI*	−0.16	9.8 × 10^−^^9^

We evaluated the genetic correlation (*r*_g_) for plausible risk factors for forearm fractures. The genetic correlations were evaluated using the LDSC tool^28^ and publicly available GWAS summary statistics (FN-BMD (ref. ^11^); LS-BMD (ref. ^11^); FA-BMD (ref. ^11^); eBMD (ref. ^10^); age at menopause^29^; age at menarche^30^; relative age at voice broke, http://www.nealelab.is/uk-biobank; grip strength^31^; falls^32^; vitamin D^33^; coronary artery disease^34^; rheumatoid arthritis^35^; inflammatory bowel disease^36^; type 2 diabetes^37^; smoking status^38^; alcohol consumption^38^; height^39^ and BMI^40^). LDSC reports an estimate of *r*_g_, together with the *P* value from a two-sided *z* test based on *r*_g_ and its standard error. For diseases/traits including UK Biobank in the GWAS and displaying statistically significant genetic correlations (*P* < 0.0028, Bonferroni correction accounting for 18 risk factors) with forearm fractures, we performed sensitivity analyses excluding UK Biobank in the forearm fracture meta-analysis used for the correlations (analyses excluding UK Biobank: eBMD, *r*_g_ = −0.62, *P* = 4.3 × 10^−^^61^; Falls, *r*_g_ = 0.23, *P* = 2.8 × 10^−^^8^). *P*, unadjusted *P* values from a two-sided *z* test.

*Statistically significant at Bonferroni-adjusted level (*P* < 0.0028).

### Causal risk factors for forearm fractures

**Fig. 4:**
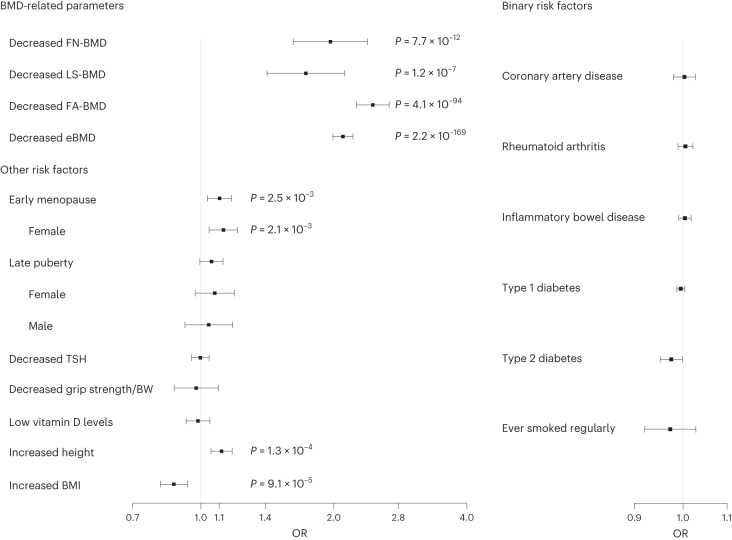
MR to estimate the causal associations for 17 genetically determined risk factors on forearm fracture risk. Data are given as OR with 95% CIs estimated using IVW MR. OR for the risk of fracture are given per s.d. change in the risk factor for continuous trait or risk of fracture per doubling of odds (obtained by multiplying the causal estimate of log odds by ln_2_ ≈ 0.693) (ref. ^41^) of disease susceptibility for binary factors. For menopause and puberty, we used the estimated s.d. from the largest cohorts in the published GWAS (early menopause s.d. = 3.81 in Breast Cancer Association Consortium^29^; late puberty s.d. = 1.40 in Women’s Genome Health Study^30^) to translate the effect unit from year to s.d. For ever smoked regularly, ORs are expressed per unit increase in log odds of ever smoking regularly with a 1 s.d. increase in genetically predicted smoking initiation corresponding to a 10% increased risk of smoking^38,42^. Grip strength is given as grip strength per BW and s.d. for grip strength is given for kg grip strength per kg in BW and was estimated in the UK Biobank to be 0.127. *P* < 0.0029 (Bonferroni correction accounting for 17 tests) was considered statistically significant. Findings with a nonadjusted *P* value (based on a two-sided *z* test) below 0.0029 have their corresponding *P* value represented in the figure. For risk factors including UK Biobank in the GWAS and displaying statistically significant causal effects with forearm fractures, we performed sensitivity analyses excluding UK Biobank in the forearm fracture meta-analysis used for the MR, revealing essentially similar effect estimates (results excluding UK Biobank in the forearm fracture GWAS; decreased eBMD OR = 2.07; 95% CI, 1.96–2.20; *P* = 1.0 × 10^−^^137^; early menopause OR = 1.10; 95% CI, 1.02–1.18; *P* = 1.5 × 10^−^^2^).

We used two-sample MR to test the causal effect of 17 plausible risk factors on forearm fractures (Fig. 4, Supplementary Fig. 8 and Supplementary Tables 19 and 20). There was clear evidence of strong causal associations of genetically decreased FA-BMD (odds ratio (OR) per s.d. decrease 2.45, 95% confidence interval (CI) 2.25–2.67), eBMD (OR per s.d. decrease 2.10, 95% CI 1.99–2.21), FN-BMD (OR per s.d. decrease 1.96, 95% CI 1.62–2.38), and LS-BMD (OR per s.d. decrease 1.73, 95% CI 1.41–2.10) with risk of forearm fracture. Among the clinical risk factors, we found that increased height (OR per s.d. increase 1.11, 95% CI 1.06–1.16) and early menopause (OR per s.d. decrease 1.12, 95% CI 1.04–1.21) were causally associated with increased risk of forearm fracture, while increased BMI was associated with reduced risk of forearm fractures (OR per s.d. increase 0.87, 95% CI 0.81–0.93). None of the other evaluated risk factors was statistically significantly causally associated with forearm fractures (Bonferroni correction accounting for 17 tests, *P* < 0.0029; Fig. 4). See Supplementary Note 5 for sensitivity analyses using alternative MR methods.

### Height as a causal risk factor for fractures at different bone sites

To determine whether height is a causal risk factor not only for forearm fractures, but also for fractures at other bone sites, we evaluated the causal associations for height with fractures at different bone sites in UK Biobank. We observed that increased height was causally associated with forearm fractures (OR 1.14; 95% CI, 1.05–1.23 per s.d. increase in height), hip fractures (OR 1.31; 95% CI, 1.17–1.47) and major osteoporotic fracture (MOF; OR 1.15; 95% CI, 1.07–1.23) but not with fractures at the lower leg (OR 1.05; 95% CI, 0.97–1.14; Supplementary Table 22).

## Discussion

We performed a large-scale GWAS meta-analysis on forearm fractures followed by replication, including more than 100,000 forearm fracture cases, and identified 43 loci, including 26 new loci, associated with forearm fractures. Although most of these exert their effects mainly via regulation of BMD, we also identified fracture loci primarily regulating bone quality parameters. Some bone-site-specific fracture signals were identified with a major impact on forearm fractures but without any association with hip fractures. MR identified tall stature and low BMI as new causal risk factors for forearm fractures. The advantage of using forearm fractures as outcome in GWAS is discussed in Supplementary Note 6.

The present study demonstrates that there are clear bone-site-specific differences for some of the identified forearm fracture loci. This was most striking for the strongest forearm fracture signal (rs2908007), at *WNT16*, that displayed remarkable bone-site-specificity with no tendency of an association with hip fractures. This genetic signal at *WNT16* has been described previously as the most pronounced signal for fractures at any bone site^10,12^, and subsequent mechanistic studies revealed that osteoblast-derived WNT16 protects against fractures in mice^22^. Our present finding indicates that the genetic association signal at the *WNT16* locus with fractures at any bone site is driven mainly by a large effect size on forearm fractures and suggests that treatments targeting WNT16 may exert bone-site-specific effects, with protective effects on forearm fractures but possibly no protective effect on hip fractures. We propose therefore that bone-site-specific fracture patterns need to be evaluated for fracture signals identified by GWAS on forearm fractures and on fractures at any bone site. When doing so, we suggest that selecting loci as potential therapeutic targets that also impact hip fractures will provide the highest clinical yield, given the medical and economic burden of these fractures. The A allele of rs2908007 at the *WNT16* locus, strongly associated with increased forearm fracture risk, was also strongly associated with reduced FA-BMD (beta, −0.14 s.d. per A allele)^11^, and its effect size for FA-BMD was approximately 3.5 times larger than its corresponding effect size for FN-BMD (beta, −0.04 s.d. per A allele)^11^. The larger effect size for FA-BMD compared with FN-BMD is most probably an important underlying factor for the observed strong association with forearm fractures but not hip fractures for this *WNT16* signal. In addition, it is possible that other described effects of WNT16 on bone-site-specific cortical bone dimensions or bone microstructure might contribute to the pronounced effect on forearm fractures^22,23^.

In the present study, most of the identified forearm fracture signals were GWS associated with eBMD. After removal of the genetic influence of eBMD, we identified genetic signals in nine loci (*EN1*, *FGFRL1*, *RSPO3*, *WNT16*, *ZNF800*, *TNFRSF11B*, *ABR*, *SOST*, *TAC4*) associated with forearm fracture risk. Two identified signals in the eBMD conditional analyses (at *ZNF800* and *ABR*) were not identified in the main forearm fracture GWAS at GWS level. Interestingly, the identified genetic variant at the *ZNF800* locus is a missense SNP (Pro103Ser) of ZNF800, suggesting that *ZNF800* could be the causal gene for this signal. The Pro103Ser variant in ZNF800 changes the amino acid sequence of the CH2 zinc finger protein—a putative transcription factor^24,25^. The fracture-reducing Ser103 allele was shown previously to associate with increased appendicular lean mass and reduced serum leptin levels^25^. The mechanism for this genetic signal to reduce forearm fracture risk is unknown but seems not to be mediated via BMD.

We identified genetic signals at three loci (*TENT5A*, *PRKAR1B* and *TAC4*) associated with forearm fractures fulfilling the predefined criteria of being a fracture locus and not being a known GWS BMD or fracture locus. The first of these three fracture loci is at *TENT5A*, which encodes the TENT5A protein—an active cytoplasmic poly(A) polymerase^17^. Both *Tent5a*^*–/–*^ mice and humans with mutations in *TENT5A* develop multiple fractures^17,18^. The underlying mechanism includes altered posttranscriptional polyadenylation with an effect on osteoblast physiology and collagen production. These studies involving disruption of TENT5A function strongly suggest that altered function of TENT5A is the underlying mechanism for the genetic signal (rs915125) at the *TENT5A* locus to regulate forearm fracture risk. The second fracture locus, not reported to be a BMD locus, is at *PRKAR1B*. The causal gene and the underlying mechanism for this forearm fracture signal remain to be determined.

The third fracture locus, not reported to be a BMD locus, is at *TAC4*. Previous studies have shown that HK1, encoded by the *TAC4* gene, inhibits substance P-induced stimulation of osteoclast formation and function in vitro, and that HK1 immunoreactivity is observed in osteocytes and osteoclasts in bone^20^. Similarly, we observed in the present study relatively high *Tac4* mRNA expression in both trabecular and cortical bone, and in situ hybridization of bone revealed high expression of *Tac4* mRNA in osteoblast-lineage cells and modest expression in osteoclasts in mice. Our functional studies demonstrated that the outer cortical bone dimensions of the long bones as well as the trabecular bone microstructure were affected in *Tac4*^–/–^ mice. We believe that both the reduced cortical bone dimensions and the impaired trabecular bone microstructure may contribute to reduced bone strength in *Tac4*^–/–^ mice. The lack of statistically significant effect on aBMD analyzed by DXA in *Tac4*^–/–^ mice may be because DXA cannot identify effects on cortical bone dimension or trabecular bone microstructure. These findings strongly suggest that *TAC4* is the causal gene for the forearm fracture signal at the *TAC4* locus. However, further studies are warranted to determine the age-dependent change in BMD in the whole skeleton and of trabecular and cortical bone parameters in *Tac4*^–*/*–^ mice. We observed a reduction in bone formation in 12-month-old *Tac4*^–/–^ mice, and this may contribute to the observed phenotype, but further studies are required to determine the mechanism underlying the reduced bone strength in *Tac4*^–/–^ mice (Supplementary Note 7).

Similarly, as has been described for fractures at any bone site^12^ and hip fractures^13^, the present study demonstrated that low BMD is the main causal risk factor for forearm fractures. Low BMI is also a known risk marker of increased fracture risk, already included in fracture prediction tool FRAX, but the possible causal association of BMI with fracture risk has previously not been investigated. We observed that low BMI and tall stature were independently estimated to be causal for forearm fracture risk. The direct causal association of height with forearm fractures is in line with observational associations demonstrating increased fracture risk in tall individuals^26^. It has been proposed that the secular trend of increased fracture incidence is the result of a concomitant secular increase of height in Norway^26^ and that the underlying mechanism might be that increased height gives more force in falls. A role of height mediated via the mechanical force imparted by a fall upon fracture risk is supported by our findings that height was causally associated with forearm and hip fractures but not with fractures of the lower leg. In general, high BMI is observationally associated with reduced fracture risk^26^, which is in line with the identified inverse causal association with forearm fractures in the present study. BMI, but not height, is already included as a clinical risk factor in the fracture prediction tool FRAX (ref. ^27^). As we demonstrate herein that height, independently of BMI, is a causal risk factor for fractures, we propose that height is a strong candidate to be included as a risk factor in the ongoing update of FRAX (ref. ^27^). This is further supported by our findings that height was causally associated not only with forearm fractures but also with hip fractures and MOF—the two fracture groups used as outcomes in the FRAX estimation. The functional annotation and pathway analyses for the identified forearm fracture signals are discussed in Supplementary Note 8.

The present study has several strengths but also limitations. A main strength is the high number of individuals with forearm fractures included in both the discovery and replication phases. Second, we focused on a single fracture site, which afforded the opportunity to identify more specific signals at higher statistical power. Moreover, as the forearm fracture GWAS was not adjusted for height and weight, we could assess the possible causal associations of BMI and height with forearm fractures. Some limitations of the present study need to be considered. As the available genetic instruments for falls and alcohol consumption were very weak, the causal associations for these two relevant risk factors for forearm fractures could not be evaluated. In addition, it is a principal limitation that the analyses in our study were restricted mainly to participants of European ancestry. Therefore, additional analyses are necessary to investigate whether our results also apply to other ancestry groups.

In conclusion, this GWAS meta-analysis identified 43 loci that were reproducibly associated with forearm fractures. Most of these loci exert their effects mainly via regulation of BMD. However, we also identified fracture loci primarily regulating bone quality parameters such as collagen fiber composition, cortical bone dimensions and trabecular bone microstructure. Some bone-site-specific fracture signals were identified, and this specificity should be considered when selecting potential novel drug targets and genetic predictors of fracture risk. Finally, tall stature and low BMI are novel causal risk factors for fractures, and we propose that height is a strong candidate to be included as a new risk factor in the ongoing update of the fracture prediction tool FRAX (ref. ^27^).

## Methods

### Participants and setting

We performed a GWAS discovery meta-analysis followed by a replication analysis for forearm fractures. For the discovery meta-analysis, we included participants from five Northern European biobanks (The Trøndelag Health Study (HUNT) from Norway, UK Biobank from UK, Umeå Fracture and Osteoporosis (UFO) from Sweden, Estonian Biobank (EstBB) from Estonia and FinnGen from Finland) with forearm fracture data and genotype data available. To reduce potential bias due to population stratification, we restricted the analyses to studies with participants of European descent in HUNT, UK Biobank, UFO and EstBB. In total, 50,471 forearm fracture cases and 969,623 controls were included in the discovery meta-analysis. Replication analyses of GWS signals were performed in three other large biobank samples (deCODE from Iceland, the Copenhagen Hospital Biobank study on osteoporosis and fractures (CHB-OF) and the Danish Blood Donor Study (DBDS) from Denmark), including 49,555 forearm fracture cases and 620,360 controls. In total, 100,026 forearm fracture cases and 1,589,983 controls were included in the discovery and replication analyses. For information on the eight contributing biobanks, see Supplementary Tables 1–4. All contributing research studies were approved by the relevant institutional ethics review boards (Supplementary Tables 1, 3 and 4).

### Forearm fracture definition

For the discovery meta-analysis using the UFO, HUNT, UK Biobank, EstBB and FinnGen cohorts, we included forearm fractures derived from high quality national registers based on medical and/or radiological reports classified according to International Classification of Diseases (ICD, corresponding to ICD10 codes S52 and ICD9 code 813). Only forearm fracture cases in patients >30 years old were included. For UK Biobank, but not for the other cohorts, we also included self-reported forearm fractures reported at the baseline visit (*n* = 6,555). Controls were defined as individuals from the same cohorts, without a history of forearm fracture.

For replication in deCODE, CHB-OF and DBDS sample sets, forearm fractures were defined by ICD codes from medical records (ICD10 codes S52 and ICD9 code 813 in the Icelandic samples, and ICD10 S52 and ICD8 N813 in the Danish samples). Controls were defined as individuals from the same cohorts without a history of forearm fracture (Supplementary Tables 1–4).

### GWAS and meta-analysis

Genome-wide genotyping was performed in each of the discovery cohorts using Illumina or Affymetrix genome-wide genotyping chips, and imputation was performed to ensure accurate ascertainment of nearly all common genetic variants above a minor allele frequency (MAF) threshold of 1% (Supplementary Table 2). We followed a standardized analytical plan to assess the association of SNPs with risk of forearm fracture in each participating cohort. Logistic models using SAIGE or PLINK software were used to estimate the SNP associations with forearm fracture, testing additive (per allele) genetic effects. The analysis was adjusted for sex, age (simple and quadratic terms), principal components, study site (when necessary) and family structure (where feasible). Study designs for the eight included cohorts are presented in Supplementary Tables 1–4. When needed, individual GWAS summary results were corrected for population stratification by the genomic control inflation factor before we performed fixed effect inverse-variance weighted (IVW) meta-analysis using the METAL software. A total of 8,396,745 autosomal and X-linked SNPs present in at least three discovery studies were meta-analyzed in the discovery stage. We standardized the genomic coordinates to be reported on the NCBI build 37 (hg19) (https://www.ncbi.nlm.nih.gov/datasets/genome/GCF_000001405.13/).

### Multi-SNP-based conditional and joint association analysis

To identify conditionally independent SNPs from this GWAS, we used GCTA-COJO (refs. ^43,44^), which leverages correlation estimates (LD) between SNPs together with summary statistics from the GWAS. The following parameters were used for COJO analyses: --MAF 0.01, --cojo-p 5e-8, --cojo-wind 10000, --cojo-collinear 0.9. SNPs with a COJO-adjusted *P* < 5 × 10^−^^8^ were considered as GWS conditionally independent signals and were selected for replication. We defined fracture-associated loci as ±500-kb windows centered on each COJO independent GWS variant. Overlapping loci were merged where the two neighboring independent GWS variants were within 500 kb of each other.

### Assessment of novelty of GWAS findings

We evaluated whether our identified replicated forearm fracture signals were associated with fractures at any bone site, hip fractures, eBMD, FN-BMD, LS-BMD, FA-BMD or total body BMD as reported in previous publicly available GWAS summary statistics^8–13^. Our identified forearm fracture signals were reported to be a new fracture locus if it or any linked SNP (*r*^2^ > 0.8) was not previously reported to be GWS for fractures^10,12,13^. Our identified forearm fracture signal was reported to be a new bone trait locus if it or any linked SNP (*r*^2^ > 0.8) was not previously reported to be GWS for fractures, eBMD, FN-BMD, LS-BMD, FA-BMD or total body BMD^8–13^. For these evaluations, we used the online available databases Phenoscanner (http://www.phenoscanner.medschl.cam.ac.uk/), Musculoskeletal knowledge portal (https://msk.hugeamp.org/) and GWAS Catalog (https://www.ebi.ac.uk/gwas/home). SNP linkage was calculated using the R package LDlinkR (ref. ^45^).

### Removal of the genetic influences of eBMD on forearm fracture risk

We aimed to identify loci that increased forearm fracture risk but not through decreased BMD. We used summary statistics of the well-powered GWAS for eBMD (ref. ^10^) and the present GWAS meta-analyses on forearm fractures in these analyses using the mtCOJO tool^46^ in the GCTA software package. A European ancestry random subset (*n* = 5,000) from UK Biobank was used as LD reference population. mtCOJO requires only summary data to conduct a GWAS analysis for one phenotype conditioned on other phenotypes.

### Functional annotation

To characterize the genetic association signals of forearm fractures, we used Functional Mapping and Annotation of GWAS (FUMA, http://fuma.ctglab.nl). FUMA is an integrative web-based platform containing information from 18 biological data repositories and tools. The FUMA pipeline has been described in detail elsewhere^47^. To search for causal genes associated with risk of forearm fracture at each locus, we annotated the identified forearm fracture signals and correlated variants (*r*^2^ > 0.8). To determine whether these SNPs are predicted to affect coding or splicing of a protein, we categorized their functional consequence using ANNOVAR (ref. ^48^). To determine whether these SNPs had statistically significant cis-eQTLs, we evaluated all available tissues in GTEx v.8 (https://www.gtexportal.org/home/). In addition, the presence of possible cis-eQTLs in osteoclasts was determined using a publicly available dataset (http://www.gefos.org/?q=content/human-osteoclast-eqtl-2018-2020). This dataset is a single cohort eQTL study of 158 human osteoclast-like cell cultures that were differentiated from peripheral blood mononuclear cells^49^. We also evaluated cis-eQTLs in primary human osteoblasts^50^. Trabecular bone samples for primary human osteoblast cultures used to develop this osteoblast dataset were collected from 95 donors who underwent total hip replacement.

Colocalization was performed to assess whether forearm fracture and tissue-specific expression of each gene shared the same causal genetic variants. We adopted the PWCoCo software with default prior settings: *P*_1_ = 1.0 × 10^−4^, *P*_2_ = 1.0 × 10^−4^ and *P*_12_ = 1.0 × 10^−5^, where *P*_1_ and *P*_2_ represent the prior probability of a variant being causally associated with forearm fracture and tissue-specific gene expression, respectively, and *P*_12_ represents the prior probability of a variant being causally associated with both traits^51,52^. Colocalization analyses leveraged summary statistics from forearm fracture GWAS and tissue-specific eQTL studies of all variants located in a ±500-kb window around each conditionally independent lead variant. A random subset of 5,000 unrelated European ancestry individuals from the UK Biobank was used as the LD reference panel. A colocalization probability (PWCoCo.H4) >0.8 was considered strong evidence of colocalization, and a PWCoCo.H4 >0.5 was considered suggestive evidence of colocalization.

We estimated the deleteriousness of the identified SNPs using Combined Annotation Dependent Depletion (CADD v.1.4) score^53^. For all SNPs highly correlated (*r*^2^ > 0.8) with the identified forearm fracture signals, we estimated their DNA features and regulatory elements in noncoding regions of the human genome using Regulomedb v.1.1 (http://www.regulomedb.org/)^54^. To determine chromatin accessibility for the 50 identified forearm fracture signals, we evaluated ATAC-seq in different bone cells using the publicly available ChIP-Atlas (https://chip-atlas.org/peak_browser; peaks were identified if they had a *Q* value <1.0 × 10^−5^) (ref. ^10^). Based on the genes at the identified loci, we also performed a gene-set enrichment analysis as implemented by the FUMA SNP2GENE function. To gain an overview of which biological pathways are involved, we used PASCAL enrichment analyses^55^ to infer enrichment of KEGG, BIOCARTA and REACTOME genesets for the identified GWAS signals.

### Genetic correlation

To estimate the genetic correlation between forearm fractures and other complex traits and diseases, we used (cross-trait) LDSR (ref. ^56^) as implemented in the LD score tool LDSC available on github (ref. ^28^). This method uses the cross-products of summary test statistics from two GWASs and regresses them against a measure of how much variation each SNP tags (LD score)^57^. The LDSR analyses were restricted to HapMap3 SNPs with MAF >5% in the 1000 Genomes European reference population. We used precalculated LD scores from the same reference population (https://data.broadinstitute.org/alkesgroup/LDSCORE/).

We estimated the genetic correlation between forearm fracture risk and 18 plausible risk factors for forearm fractures. In general, the selection of plausible clinical risk factors for evaluation of genetic correlation with forearm fractures in Table 2 and for MR in Fig. 4 was similar as reported in previous GWASs on hip fractures^13^ and fractures at any bone site^12^ but with the addition of height, BMI and FA-BMD. In contrast to the previous fracture GWAS^12,13^, our GWAS did not adjust for height or weight; therefore, analyses using height and BMI as potential risk factors were feasible. Finally, as we evaluated forearm fractures, we also included FA-BMD as a plausible risk factor to be investigated. We used available GWAS summary statistics for the following traits: FN-BMD (ref. ^11^), LS-BMD (ref. ^11^), FA-BMD (ref. ^11^), eBMD (ref. ^10^), age at menopause^29^, age at menarche^30^, relative age at voice break (http://www.nealelab.is/uk-biobank), grip strength^31^, vitamin D levels^33^, falls^32^, coronary artery disease^34^, rheumatoid arthritis^35^, inflammatory bowel disease^36^, type 2 diabetes^37^, smoking status^38^, alcohol consumption^38^, height^39^ and BMI^40^. We accounted for multiple testing by using a conservative Bonferroni correction for 18 tests (*P* < 0.0028).

### MR analysis of risk factors for fractures

To assess causal effect of plausible risk factors on the risk of forearm fractures, we performed two-sample MR analyses. We used genetic instrument variables obtained from selected GWAS as proxies for FN-BMD (ref. ^8^), LS-BMD (ref. ^8^), FA-BMD (ref. ^11^), eBMD (ref. ^10^), age at menopause^12,29^, age at puberty^12,30^, thyroid-stimulating hormone (TSH)^12,58^, grip strength^31^, vitamin D levels^33^, height^39^, BMI^40^, coronary artery disease^12,59^, rheumatoid arthritis^35^, inflammatory bowel disease^12,60^, type 1 diabetes^12,61^, type 2 diabetes^12,37^ and ever smoked regularly^38^. Although alcohol consumption and falls are plausible causal risk factors for forearm fractures, these were not included in the MR analyses as the available genetic instruments were very weak, resulting in insufficient power in the analyses^12,38^. We only selected variants with a MAF >1% that were strongly associated with the clinical risk factor (*P* < 5 × 10^−^^8^), ensuring that the genetic variants used as instrumental variables are associated with the clinical risk factor. We selected instruments with *r*^2^ < 0.01 (based on the European populations in Ldlink (ref. ^62^)) to ensure that there was little correlation between instruments. As the primary MR analyses, we used combined weighted estimates by IVW using fixed or random effects depending on Cochran’s *Q* statistic test of heterogeneity. We then used the MR-Egger regression as a sensitivity analysis to test for possible directional horizontal pleiotropy. This method uses a weighted regression with an unconstrained intercept to regress the effect sizes of variant risk factor associations. It can thus detect some violations of the standard MR assumptions and provide an effect estimate that is not subject to these violations^63^. In further sensitivity analyses, we used weighted median MR. We applied a conservative Bonferroni-corrected threshold accounting for 17 tests (*P* < 0.0029).

Since height is used in the calculation of BMI (weight/height squared) and height and BMI are modestly correlated (men *r* = −0.07, women *r* = −0.17 in the UK Biobank of the participants included in the present forearm fracture GWAS), we used a multivariable MR approach to estimate their independent causal associations with forearm fractures^64^. We consider it more clinically relevant to evaluate BMI and height as possible causal clinical risk factors compared with weight and height as BMI already is included in the fracture prediction tool FRAX (ref. ^27^). According to the national guidelines in many countries, this fracture prediction tool should be used to aid in fracture risk prediction and thereby the selection of individuals who would benefit most from osteoporosis treatment^27^. To reduce the possible impact of heterogeneity introduced by the height SNPs in this multivariable MR, we also performed analyses excluding outlier of genetic instruments using MR-PRESSO (ref. ^65^) and MR-LASSO (ref. ^66^). The MR analyses were conducted using the R-packages MendelianRandomization^67^ and MR-PRESSO.

### Height as a causal risk factor for fractures at different bone sites

To estimate the effects of increased height on risk of fractures at different bone sites, we performed MR analyses on bone-site-specific fracture outcomes. SNP associations with fractures at different bone sites were estimated in UK Biobank. Fracture cases were identified using ICD10 and 9 codes (Supplementary Table 22) and included the following fracture groups: forearm fractures, hip fractures, MOF (distal forearm fractures + hip fractures + vertebral fractures + upper arm fractures) and fractures of the lower leg.

### Power calculation

Power calculations were performed to test whether our MR studies were adequately powered to detect a statistically significant change in the forearm fracture outcomes using IVW MR. For each trait, we used the variance explained by the instrument variables (*R*^2^ for continuous risk factors and available pseudo *R*^2^ for binary risk factors) either reported in the corresponding GWAS publication or estimated using the method described by Shim et al.^68^, the proportion of fracture cases and the sample size, to estimate the power to detect different ORs. Power calculations were conducted using the online tool http://cnsgenomics.com/shiny/mRnd/ (ref. ^69^).

### Animal experiments

Animal experiments were performed on female and male 12-month-old *Tac4*-deficient (*Tac4*^–/–^) and wild-type C57Bl/6 mice. The original breeding pairs of the *Tac4*^–/–^ mice were generated previously^70^. *Tac4*^–/–^ mice were generated on a C57Bl/6 background and backcrossed to homozygosity for more than five generations, and wild-type C57Bl/6 mice were used as controls. Animals were kept under a standard 12-h light/dark cycle in 50–60% humidity at a temperature of 24 ± 2 °C. Food and water were provided ad libitum in the Animal House of the Department of Pharmacology and Pharmacotherapy of the University of Pécs. All procedures were performed according to the European legislation (Directive 2010/63/EU) and Hungarian Government regulation (40/2013., II. 14.) and were approved by the National Ethics Committee on Animal Research of Hungary (license no.: BA/73/00657-3/2022).

### Rotarod analysis of motor coordination

Motor coordination was examined with the accelerating Rotarod on female and male 11-month-old Tac4-deficient (*Tac4*^–/–^) and wild-type C57Bl/6 mice^71^.

### Assessment of mouse bone parameters

#### Dual-energy X-ray absorptiometry

Analyses of whole tibia aBMD were performed ex vivo using Faxitron UltraFocus DXA (Faxitron Bioptics).

#### High-resolution µCT

High-resolution µCT was used to analyze lumbar vertebra 5 (L5, Skyscan, catalog no. 1275; Bruker MicroCT)^16^. The L5 was imaged with an X-ray tube voltage of 40 kV and a current of 200 µA, with a 1.0-mm aluminum filter. The scanning angular rotation was 180°, and the angular increment was 0.40°. NRecon (v.2.0.0.5, Bruker MicroCT) was used to perform reconstruction after scans. The trabecular bone in the vertebrae was analyzed 7.0 µm from the lower end of the pedicles and continued for 245 µm in the caudal direction.

#### Peripheral quantitative computed tomography

Peripheral quantitative computed tomography was performed on tibia using peripheral quantitative computed tomography XCT Research M (v.4.5B; Norland) at a resolution of 70 µm (refs. ^72,73^). Cortical bone was determined with a mid-diaphyseal scan positioned distal from the proximal growth plate of the tibia, corresponding to 30% of the bone length.

#### Mechanical strength

Three-point bending was performed on the tibia with a span length of 5.0 mm and a loading speed of 0.155 mm s^−^^1^ using an Instron 3366 (Instron). Biomechanical parameters, based on the recorded load deformation curves, were calculated from Bluehill Universal software v.4.25 (Instron) with custom-made Excel (Microsoft) macros^74^. Lumbar vertebra L5 was axially loaded with a press head of 0.9 mm in diameter, with a 1.9-mm-thick pin through the vertebral foramen to stabilize the sample for testing. The loading speed was 0.155 mm s^−^^1^. The maximal force was recorded using Instron 3366 testing equipment (Instron) and Bluehill Universal software v.4.25 software with custom-made Microsoft Excel macros.

### Histomorphometry

For the measurement of dynamic bone parameters, the mice were double labeled with alizarin (Merck, catalog no. A3882) and calcein (Merck, catalog no. C0875), which were injected (intraperitoneally) into the mice 2 and 14 days before necropsy, respectively. L4 vertebrae were fixed in 4% paraformaldehyde, dehydrated in 70% ethanol and embedded in methylmethacrylate. Toluidine-Blue-stained sections (5 μm thick) of L4 vertebral bodies were used to measure osteoid surface per bone surface (%) and osteoblast surface per bone surface (%), while tartrate-resistant acid phosphatase staining was performed to quantify the osteoclast surface per bone surface (%)^75^. Unstained sections (11 μm thick) were used to assess mineral appositional rate and labeled mineralizing surfaces per bone surface, which allowed us to calculate the bone formation rate. All parameters were measured using Histolab software (Microvision) following the guidelines of the American Society for Bone and Mineral Research^76^. Vertebrae were analyzed by Bioscar INSERM U1132.

### RNA isolation, cDNA synthesis and quantitative real-time PCR analyses

Total RNA was prepared from cortical bone, trabecular enriched bone from the vertebral body, hypothalamus, brain cortex, uterus, spleen, thymus, lung, gonadal fat, retroperitoneal fat, kidney, muscle, heart, liver, brown fat, bone marrow and pancreas using Trizol reagent (Thermo Fisher Scientific, catalog no. 15596018) and an RNeasy mini prep kit (Qiagen, catalog no. 74116) in a Qiacube preparation robot (Qiagen). The mRNA was reversed transcribed to cDNA (Thermo Fisher Scientific, catalog no. 4374967) and real-time PCR analyses were performed using the StepOnePlus Real-Time PCR System (v.2.3, Thermo Fisher Scientific). The following Assay-on-Demand primer and probe set detecting *Tac4* (Mm00474083_m1) was used. The relative gene expression was calculated by 2^−∆∆Ct^ method using the expression of the 18S ribosomal subunit (Thermo Fisher Scientific, catalog no. 4310893E) as internal standard.

### Multiplex FISH

Mice tibiae were fixated in 4% paraformaldehyde for 24 h and decalcified in 10% EDTA with 0.4% paraformaldehyde for 3 weeks. Tissues were embedded with paraffin and sectioned at 3-µm thickness. After deparaffinization, multiplex FISH was performed using the RNAscope Multiplex Fluorescent v.2 Assay (323100, Advanced Cell Diagnostics (ACD), Bio-Techne Ltd.) according to manufacturer’s instructions with minor modifications, as described below. Rehydrated sections were blocked by kit-provided hydrogen peroxide and heated in target retrieval buffer, then digested by 10% pepsin. FISH target probes were applied and incubated overnight at 40 °C. The following probes were used: Mm-Tac4 (ACD; catalog no. 449651), Mm-Sost (ACD; catalog no. 410031-C2), Mm-Ctsk (ACD; catalog no. 464071-C2), and Mm-Runx2 (ACD; catalog no. 414021-C2). Tyramide signal amplification plus fluorophore kits (PerkinElmer; catalog nos. NEL744001KT and NEL745001KT) were applied to develop C1 and C2 signals with the dilution factor 1:2,000, followed by DAPI counterstain. Signals were detected by a Leica SP8 confocal microscope.

### Reporting summary

Further information on research design is available in the Nature Portfolio Reporting Summary linked to this article.

## Online content

Any methods, additional references, Nature Portfolio reporting summaries, source data, extended data, supplementary information, acknowledgements, peer review information; details of author contributions and competing interests; and statements of data and code availability are available at 10.1038/s41588-023-01527-3.

### Supplementary information

Supplementary Information Consortium member lists, Supplementary Notes 1–8 and Figs. 1–8.

Reporting Summary

Supplementary Table Supplementary Tables 1–22.

## Data Availability

Summary statistics from the GWAS meta-analysis are available at the GWAS Catalog under study accession number GCST90281273 (https://www.ebi.ac.uk/gwas). For cohort-specific datasets, each individual cohort has to be contacted as each country and cohort has different data access policies. Individual-level EstBB data are available under restricted access administered by the Estonian Genome Center of the University of Tartu in accordance with the regulations of the Estonian Human Genes Research Act. Access can be obtained by application at https://genomics.ut.ee/en. Individual-level data from FinnGen participants can be accessed by approved researchers through the Fingenious portal (https://site.fingenious.fi/en/) hosted by the Finnish Biobank Cooperative FinBB (https://finbb.fi/en/). Access to UK Biobank data can be obtained by application to UK Biobank (https://www.ukbiobank.ac.uk/). Individual-level data from DBDS cohort can be accessed by contacting the steering committee (info@dbds.dk). Data access requires that projects and applicants obtain permission from the Regional Committees on Health Research Ethics and the Danish Data Protection Agency. Researchers can apply for HUNT data access from HUNT Research Centre (https://www.ntnu.edu/hunt) if they have obtained project approval from the Regional Committee for Medical and Health Research Ethics. Information on the application and conditions for data access is available at https://www.ntnu.edu/hunt/data. For cohort-specific data requests of the remaining cohorts used in the present forearm fracture study, contact U. Styrkarsdottir (unnur.styrkarsdottir@decode.is) for the deCODE cohort, U. Pettersson-Kymmer (ulrika.pettersson@umu.se) for the UFO cohort and S. Rye Ostrowski (sisse.rye.ostrowski@regionh.dk) for the CHB-OF cohort. All GWAS summary statistics used for risk factors in genetic correlations are available online: FN-BMD, LS-BMD, FA-BMD, eBMD and falls, http://www.gefos.org/; age at menopause and age at menarche, https://www.reprogen.org/data_download.html; relative age voice broke, http://www.nealelab.is/uk-biobank; grip strength, http://ldsc.broadinstitute.org/; vitamin D, levels https://cnsgenomics.com/content/data; coronary artery disease, http://www.cardiogramplusc4d.org/data-downloads/; rheumatoid arthritis, http://plaza.umin.ac.jp/~yokada/datasource/software.htm; inflammatory bowel disease, https://www.ebi.ac.uk/gwas/studies/GCST003043; type 2 diabetes, https://diagram-consortium.org/downloads.html; smoking initiation and alcohol consumption (drinks per week), https://conservancy.umn.edu/handle/11299/201564; height and BMI, https://portals.broadinstitute.org/collaboration/giant/index.php/GIANT_consortium_data_files. All look-ups were made in publicly available datasets and databases (GWAS Catalog, CADD, GTEx v.8 and Regulome DB via FUMA web application: https://fuma.ctglab.nl/; ChIP-Atlas: https://chip-atlas.org/peak_browser; previously published GWASs for FN-BMD, LS-BMD, eBMD and any fracture http://www.gefos.org/ and previously published GWAS for hip fracture https://www.ebi.ac.uk/gwas/publications/36260985).

## Code availability

All analyses have been performed using publicly available software, tools, packages and databases. SAIGE v.0.38 and PLINK v.2.0 software were used to estimate the SNP associations (https://github.com/weizhouUMICH/SAIGE and https://www.cog-genomics.org/plink2/). METAL (release March 25, 2011) software was used for fixed effect IVW meta-analysis (https://genome.sph.umich.edu/wiki/METAL). The GCTA v.1.93 software package was used to identify conditionally independent SNPs (https://yanglab.westlake.edu.cn/software/gcta/#COJO). FUMA v.1.4.1 was used to characterize the genetic association signals of forearm fracture (https://fuma.ctglab.nl/). ANNOVAR was used in FUMA to categorize the functional sequences of the SNPs. PASCAL enrichment analysis software was used to gain an overview of biological pathways (https://www2.unil.ch/cbg/index.php?title=Pascal). LDSC v.1.0.1 was used to estimate the genetic correlation between forearm fractures and other traits and diseases (https://github.com/bulik/ldsc). The MR analyses were conducted using R v.4.2.1 (https://cran.r-project.org/) and the R-packages MendelianRandomization v.0.7.0 (https://cran.r-project.org/web/packages/MendelianRandomization/index.html) and MR-PRESSO v.1 (https://github.com/rondolab/MR-PRESSO).
