# Supplementary material for: An atlas of genetic determinants of forearm fracture
Source: Nat Genet. 2023 Nov 2;55(11):1820–30. doi: 10.1038/s41588-023-01527-3 (PMC10632131; doi:10.1038/s41588-023-01527-3)
Supplement: Supplementary file 1 — Consortium member lists, Supplementary Notes 1–8 and Figs. 1–8. [file 41588_2023_1527_MOESM1_ESM.pdf]

nature genetics

Article https://doi.org/10.1038/s41588-023-01527-3

An atlas of genetic determinants of forearm fracture

In the format provided by the authors and unedited

Supplementary information

Consortium author lists

Supplementary Notes 1-8

Supplementary Figures 1-8

Consortium author lists

The DBDS Genomic Consortium collaborators

n	First name	Surname	Email	Affiliation 1	Affiliation 2
1	Karina	Banasik	karina.banasik@cpr.ku.dk	Novo Nordisk Foundation Center for Protein Research, Faculty of Health and Medical Sciences, University of Copenhagen, Copenhagen, Denmark	
2	Jakob	Bay	jabay@regionsjaelland.dk	Department of Clinical Immunology, Zealand University Hospital, Køge, Denmark	
3	Jens Kjærgaard	Boldsen	jenbol@rm.dk	Department of Clinical Immunology, Aarhus University Hospital, Aarhus, Denmark	
4	Thorsten	Brodersen	thobr@regionsjaelland.dk	Department of Clinical Immunology, Zealand University Hospital, Køge, Denmark	
5	Søren	Brunak	soren.brunak@cpr.ku.dk	Novo Nordisk Foundation Center for Protein Research, Faculty of Health and Medical Sciences, University of Copenhagen, Copenhagen, Denmark	
6	Kristoffer	Burgdorf	kristoffer.soelvsten.burgdorf@regionh.dk	Novo Nordisk Foundation Center for Protein Research, Faculty of Health and Medical Sciences, University of Copenhagen, Copenhagen, Denmark	Department of Clinical Immunology, Copenhagen University Hospital, Rigshospitalet, Copenhagen, Denmark
7	Mona Ameri	Chalmer	mona.ameri.chalmer@regionh.dk	Danish Headache Center, Department of Neurology, Copenhagen University Hospital, Rigshospitalet-Glostrup, Copenhagen, Denmark	
8	Maria	Didriksen	maria.didriksen@regionh.dk	Department of Clinical Immunology, Copenhagen University Hospital, Rigshospitalet, Copenhagen, Denmark	
9	Khoa Manh	Dinh	khoadinh@rm.dk	Department of Clinical Immunology, Aarhus University Hospital, Aarhus, Denmark	
10	Joseph	Dowsett	joseph.dowsett@regionh.dk	Department of Clinical Immunology, Copenhagen University Hospital, Rigshospitalet, Copenhagen, Denmark	
11	Christian	Erikstrup	christian.erikstrup@skejby.rm.dk	Department of Clinical Immunology, Aarhus University Hospital, Aarhus, Denmark	Department of Clinical Medicine, Health, Aarhus University, Aarhus, Denmark
12	Bjarke	Feenstra	Bjarke Feenstra <FEE@ssi.dk>	Department of Clinical Immunology, Copenhagen University Hospital, Rigshospitalet, Copenhagen, Denmark	Statens Serum Institut, Copenhagen, Denmark
13	Frank	Geller	FGE@ssi.dk	Department of Clinical Immunology, Copenhagen University Hospital, Rigshospitalet, Copenhagen, Denmark	Statens Serum Institut, Copenhagen, Denmark
14	Daniel	Gudbjartsson	Daniel.Gudbjartsson@decode.is	deCODE Genetics, Reykjavik, Iceland	
15	Thomas Folkmann	Hansen	thomas.hansen@regionh.dk	Danish Headache Center, Department of Neurology, Copenhagen University Hospital, Rigshospitalet-Glostrup, Copenhagen, Denmark	
16	Lotte	Hindhede	LOTHIN@rm.dk	Department of Clinical Immunology, Aarhus University Hospital, Aarhus, Denmark	
17	Henrik	Hjalgrim	HHJ@cancer.dk	Danish Cancer Society Research Center, Copenhagen, Denmark	Department of Epidemiology Research, Statens Serum Institut, Copenhagen, Denmark
18	Rikke Louise	Jacobsen	rikke.louise.jacobsen@regionh.dk	Department of Clinical Immunology, Copenhagen University Hospital, Rigshospitalet, Copenhagen, Denmark	
19	Gregor	Jemec	gbj@regionsjaelland.dk	Department of Clinical Medicine, Zealand University hospital, Roskilde, Denmark	
20	Katrine	Kaspersen	kathkasp@rm.dk	Department of Clinical Immunology, Aarhus University Hospital, Aarhus, Denmark	
21	Bertram Dalskov	Kjerulff	berkje@rm.dk	Department of Clinical Immunology, Aarhus University Hospital, Aarhus, Denmark	
22	Lisette	Kogelman	Lisette.kogelman@regionh.dk	Danish Headache Center, Department of Neurology, Copenhagen University Hospital, Rigshospitalet-Glostrup, Copenhagen, Denmark	
23	Margit Anita Hørup	Larsen	Margit.Anita.Hoerup.Larsen@regionh.dk	Department of Clinical Immunology, Copenhagen University Hospital, Rigshospitalet, Copenhagen, Denmark	
24	Ioannis	Louloudis	ioannis.louloudis@cpr.ku.dk	Novo Nordisk Foundation Center for Protein Research, Faculty of Health and Medical Sciences, University of Copenhagen, Copenhagen, Denmark	
25	Agnete	Lundgaard	agnete.lundgaard@cpr.ku.dk	Novo Nordisk Foundation Center for Protein Research, Faculty of Health and Medical Sciences, University of Copenhagen, Copenhagen, Denmark	
26	Susan	Mikkelsen	susanmke@rm.dk	Department of Clinical Immunology, Aarhus University Hospital, Aarhus, Denmark	
27	Christina	Mikkelsen	christina.mikkelsen@regionh.dk	Department of Clinical Immunology, Copenhagen University Hospital, Rigshospitalet, Copenhagen, Denmark	
28	Kaspar Rene	Nielsen	k.nielsen@rn.dk	Department of Clinical Immunology, Aalborg University Hospital, Aalborg, Denmark	Department of Clinical Medicine, Aalborg University, Aalborg, Denmark
29	Ioanna	Nissen	ioanna.nissen@regionh.dk	Department of Clinical Immunology, Copenhagen University Hospital, Rigshospitalet, Copenhagen, Denmark	
30	Mette	Nyegaard	nyegaard@hst.aau.dk	Department of Health Science and Technology, Faculty of Medicine, Aalborg Univeristy, Aalborg, Denmark	
31	Sisse Rye	Ostrowski	sisse.rye.ostrowski@regionh.dk	Department of Clinical Immunology, Copenhagen University Hospital, Rigshospitalet, Copenhagen, Denmark	Department of Clinical Medicine, Faculty of Health and Medical Sciences, University of Copenhagen, Copenhagen, Denmark
32	Ole Birger	Pedersen	olbp@regionsjaelland.dk	Department of Clinical Immunology, Zealand University Hospital, Køge, Denmark	Department of Clinical Medicine, Faculty of Health and Medical Sciences, University of Copenhagen, Copenhagen, Denmark
33	Alexander	Pil Henriksen	alexander.henriksen@cpr.ku.dk	Novo Nordisk Foundation Center for Protein Research, Faculty of Health and Medical Sciences, University of Copenhagen, Copenhagen, Denmark	
34	Palle Duun	Rohde	palledr@hst.aau.dk	Department of Health Science and Technology, Faculty of Medicine, Aalborg Univeristy, Aalborg, Denmark	
35	Klaus	Rostgaard	klar@cancer.dk	Danish Cancer Society, Copenhagen, Denmark	Department of Epidemiology Research, Statens Serum Institut, Copenhagen, Denmark
36	Michael	Schwinn	michael.schwinn@regionh.dk	Department of Clinical Immunology, Copenhagen University Hospital, Rigshospitalet, Copenhagen, Denmark	
37	Kari	Stefansson	kari.stefansson@decode.is	deCODE Genetics, Reykjavik, Iceland	
38	Hreinn	Stefánsson	hreinn.stefansson@decode.is	deCODE Genetics, Reykjavik, Iceland	
39	Erik	Sørensen	Erik.Soerensen@regionh.dk	Department of Clinical Immunology, Copenhagen University Hospital, Rigshospitalet, Copenhagen, Denmark	
40	Unnur	Þorsteinsdóttir	Unnur.Thorsteinsdottir@decode.is	deCODE Genetics, Reykjavik, Iceland	
41	Lise Wegner	Thørner	Lise.Wegner.Thoerner@regionh.dk	Department of Clinical Immunology, Copenhagen University Hospital, Rigshospitalet, Copenhagen, Denmark	
42	Mie	Topholm Bruun	mie.topholm.bruun@rsyd.dk	Department of Clinical Immunology, Odense University Hospital, Odense, Denmark	
43	Henrik	Ullum	HEUL@ssi.dk	Statens Serum Institut, Copenhagen, Denmark	
44	Thomas	Werge	thomas.werge@regionh.dk	Institute of Biological Psychiatry, Mental Health Centre, Sct. Hans, Copenhagen University Hospital, Roskilde, Denmark	Department of Clinical Medicine, Faculty of Health and Medical Sciences, University of Copenhagen, Copenhagen, Denmark
45	David	Westergaard	david.westergaard@cpr.ku.dk	Novo Nordisk Foundation Center for Protein Research, Faculty of Health and Medical Sciences, University of Copenhagen, Copenhagen, Denmark	

The Estonian Biobank Research Team

Andres Metspalu, Lili Milani, Tõnu Esko, Reedik Mägi, Mari Nelis, Georgi Hudjashov

Supplementary Notes 1-8

Supplementary Note 1

eBMD and the different aBMDs are major factors underlying forearm fracture risk for most of the identified forearm fracture loci

For 39 out of the 43 identified forearm fracture loci, the identified top signal, or a linked SNP ($r^2>0.8$), was previously reported to be associated with eBMD at GWS level in the expected direction (the allele associated with increased fracture risk was associated with reduced eBMD; Table 1).[1] For 22 of the 43 identified forearm fracture loci, the identified signal, or a linked SNP ($r^2>0.8$), was previously reported to be associated with any of the four aBMD parameters analyzed using DXA (FN-BMD, LS-BMD, FA-BMD, or total body BMD) at GWS level in the expected direction (the allele associated with increased fracture risk was associated with reduced aBMD, Table 1)[2-4] These data demonstrate that eBMD and the different aBMDs are major factors underlying forearm fracture risk for most of the identified forearm fracture loci. They also suggest that some loci act upon fracture without major influence on BMD.

Candidate approach starting from known BMD signals

In a candidate approach, we next evaluated the associations for GWS top SNPs from a previous published eBMD GWAS[1] with forearm fractures using the present forearm fracture GWAS meta-analysis. We observed that 217 of 512 GWS top signals were also associated with forearm fracture risk (217 passing nominal significance $p < 0.05$ and 76 passing Bonferroni-adjusted significance $p < 9.7\times10^{-5}$) in the expected direction (the allele associated with decreased eBMD was associated with increased forearm fracture risk; Supplementary Table 6). Similarly, 31 of 43 (31 passing nominal significance $p < 0.05$ and 21 passing conservative Bonferroni-adjusted significance $p<0.0012$) FN-BMD GWS top signals and 28 of 38 (28 passing nominal $p < 0.05$ and 19 passing conservative Bonferroni-adjusted $p < 0.0013$) LS-BMD GWS top signals[2] were also associated with risk of forearm fracture in the expected direction. These findings demonstrate that many of the identified eBMD and aBMD signals associate with forearm fracture risk. Further, a majority of the 50 identified forearm fracture signals are reported to be mainly associated with BMD-related parameters when evaluated in the GWAS catalog (Supplementary Table 7).

Supplementary Note 2

Functional annotation, eQTLs and pathway analyses

To search for causal genes associated with risk of forearm fracture, we annotated the 50 identified forearm fracture signals and correlated variants (r^2>0.8) with regards to their functional consequences. Signals in 2 of these loci were predicted to affect coding (missense) of a protein (Supplementary Table 8), including an amino acid substitution in LRP5 (rs4988321, Val667Met). Signals in 26 loci had at least one significant cis-eQTL according to GTEx V8 (Supplementary Table 9, false discovery rate < 5%). Furthermore, we observed that the forearm fracture signal in the *TAC4* locus is associated with the expression of a *TAC4* antisense (RP11-304F15.3; $p=2.6x10^{-8}$, in blood (https://www.eqtlgen.org/). In addition, the forearm fracture signal in the *PRKAR1B* locus is robustly associated with the expression of an antisense to *PRKAR1B* (antisense AC147651.4, $p=2.0\times10^{-28}$ in blood; GTEx V8). Finally, using MR it has been reported that circulating RSPO3 is causally associated with forearm fractures.[5] Of all these signals that were predicted to be coding, having at least one cis-eQTL, or having strong plasma protein MR support (RSPO3 with MR support from a previously published report[5]) for causality on forearm fractures, 9 had evidence that pointed to a single gene for each locus (Supplementary Figure 3). Among these, there was evidence of colocalization between forearm fractures and cis-eQTLs for 4 signals, including strong evidence (PWCoCo.H4 >0.8) for *PRKCE*, *GREM2*, and *B4GALNT3*, and suggestive evidence (PWCoCo.H4 =0.75) for *RSPO3* (Supplementary Table 9). Additionally, using a publicly available data set of osteoclast eQTLs, we identified one cis-eQTL for rs4671960 (*SPTBN1*, $p = 4.8 \times 10^{-9}$, cis-eQTLs effect allele G, Beta 0.64 of normalized gene expression per effect allele)[6] with strong colocalization evidence (PWCoCo.H4 = 0.97). Using a data set of osteoblasts, analyses identified that the forearm fracture signal rs113061374 was associated with two separate transcripts, thereby not pointing to one specific underlying gene (rs113061374 cis-eQTLs; *UVSSA*, $p= 5.4 \times 10^{-8}$; *CRIPAK* $p= 5.3 \times 10^{-7}$).

We estimated the deleteriousness of the 50 variants with a scaled Combined Annotation Dependent Depletion (CADD) score[7], which ranks variants based on predicted pathogenicity. We found that 8 variants had a CADD score >12.37, which is a suggested threshold for pathogenicity from a previous report (Supplementary Table 10)[8] but two of them had cis-eQTLs pointing to at least two different genes (Supplementary Table 9; Supplementary Figure 3). Furthermore, Regulome DB (RDB) scores that provide evidence that the signal is located in a functional region revealed that the lead SNP, rs4671960, at the *SPTBN1* locus had a RDB

score of 1f (likely to comprise a DNA regulatory element and linked to expression of a gene target, Supplementary Table 11). As described above, this SNP is also a significant cis-eQTL for *SPTBN1* in osteoclasts. In addition, a proxy SNP, rs4647934 (r^2=0.99), to a lead SNP (rs78520297) at the *FGFRL1* locus as well as two proxy SNPs (rs17679475, rs2273703, both with r^2=0.94) to the lead SNP (rs1467561) at the *TMRT61A* locus had RDB scores of 1f. The lead SNP at the *TAC4* locus (rs79049182), associated with the expression of a *TAC4* antisense, had a RDB score of 2b (likely to comprise a DNA regulatory element, Supplementary Table 11). To determine chromatin accessibility for the 50 identified forearm fracture signals, we evaluated ATAC-seq in different types of bone cells. We found evidence of chromatin accessibility by ATAC-seq peak for at least one of the available bone cell types for 10 (20%) of the 50 conditionally independent lead variants (Supplementary Table 12). In contrast, out of 129,699 tested variants located within 500kb of any lead variants, 9798 (7.5%) overlapped with ATAC-seq peak for at least one bone cell type, whereas only 5.5% (456,518 out of 8,266,996) of tested variants located more than 500kb away from the lead variants showed such evidence. Thus, the lead variants demonstrated a 3.06-fold enrichment in open chromatin regions compared to variants located within 500kb (95% CI 1.36-6.23; Fisher's exact test p-value = $3.8x10^{-3}$), and a 4.28-fold enrichment compared to variants located more than 500kb away (95% CI 1.91-8.71; Fisher's exact test p-value = $3.5x10^{-4}$).

Based on the genes at the 43 loci, we also performed a gene-set enrichment analysis in FUMA and found a strong enrichment for genes associated with eBMD and different aBMD parameters and for genes involved in WNT signaling pathways. In addition, we observed a moderate enrichment for genes associated with systolic blood pressure (Supplementary Table 13). To gain an overview of which biological pathways are involved, we also used PASCAL enrichment analyses[9] to infer enrichment of KEGG, BIOCARTA, and REACTOME gene sets for the identified GWAS signals. These analyses revealed enrichment of several bone-related pathways, such as WNT signaling, Hedgehog signaling, and TGF-beta signaling pathways (Supplementary Figure 4).

Supplementary Note 3

TAC4 expression

When comparing with the expression in multiple human tissues (n=54) available at the GTEx portal (https://gtexportal.org/home/), the expression of *TAC4* was highest in the pituitary (Supplementary Figure 6C) but bone was not included in that panel. We compared the *Tac4*

expression in 18 mouse tissues and observed the highest levels in the spleen, the second highest levels in trabecular enriched vertebral body bone, and the third highest in cortical bone (Supplementary Figure 6D). To determine the localization of the cells expressing *Tac4* within the skeleton, fluorescence in situ hybridization was performed (Fig 3, Supplementary Figure 7). *Tac4* was present in *Runx2*-expressing osteoblasts on the trabecular and cortical bone surfaces and in *Sost*-expressing osteocytes (Fig. 3; Supplementary Figure 7). In addition, *Tac4* mRNA expression was modestly detected in *Ctsk*-expressing osteoclasts in both trabecular and cortical bone (Fig 3; Supplementary Figure 7).

Supplementary Note 4

Genetic correlation between forearm fractures and measures of bone size

As bone size also may influence fracture risk, supported by the observed reduced cortical bone width in the *Tac4*$^{-/-}$ mice, we made additional exploratory analyses determining the genetic correlation of forearm fractures with different measures of bone size from a previous GWAS[10] based on five different bone areas in the hip and lumbar spine regions determined by DXA. However, no significant genetic correlations were observed for any of these bone areas (r_g between -0.05 to 0.08 and p-values between 0.61-0.13; Supplementary Table 18). This suggests that these measures of bone size and forearm fracture do not share a common genetic etiology.

Supplementary Note 5

Sensitivity analyses using alternative MR methods

For the reported significant causal associations except for eBMD, we observed no evidence of horizontal pleiotropy between the instruments and the outcomes using MR Egger regression, and the estimates from the IVW meta-analyses were, in general, very similar to the estimates from the weighted median and penalized weighted median methods (Supplementary Table 20; Supplementary Figure 8). For eBMD, the Egger estimate was similar to the estimate from IVW (Supplementary Table 20, Supplementary Figure 8).

As height and BMI (weight/(height)^2) are related, we determined their independent causal associations with forearm fractures by including both these risk factors as exposures in a multivariable MR model. Using a variety of MR methods, also including MR-PRESSO and MR-LASSO to exclude outlier of genetic instruments for height, we observed that increased

height and reduced BMI were both independently causally associated with increased risk of forearm fractures, with very similar effect estimates as observed in the univariate MR analyses (Supplementary Table 21).

Supplementary Note 6

The advantage of using forearm fractures as outcome in GWAS

Previous GWAS on BMD parameters have identified multiple genetic BMD signals[1-4], and MR studies have revealed that low BMD is a strong causal risk factor for combined fractures at many different bone sites[11] and for hip fractures.[12] Thus far, the largest GWAS on fractures at any bone sites identified 15 independent fracture loci, and all these were BMD-dependent fracture signals.[11] As the heritable component of fracture risk is proposed to be partly independent of BMD,[13,14] we hypothesized that the mechanisms for fractures at different bone sites might differ and that the major common causal risk factor for fractures at different bone sites is low BMD. Therefore, bone site-specific BMD-independent fracture signals might exist, but the strength of these will be diluted in combined GWAS of fractures at multiple different bone sites. The present identification of three fracture loci, not being known BMD loci, is proof of concept that the likelihood of identifying fracture loci, not only having effect via BMD, is enhanced when only evaluating forearm fractures, a well-defined fracture type that occur rather early in life when fracture heritability is high.[15] We propose that the early age of forearm fractures, prior to acquisition of major age-dependent BMD changes, may enhance the likelihood of identifying non-BMD fracture signals with impact on bone site-specific cortical bone dimensions, trabecular bone microstructure, and other bone quality parameters.

Supplementary Note 7

Discussion on eBMD in relation to the *TAC4* locus

Removal of the genetic influence of eBMD did not affect the association between the genetic signal at the *TAC4* locus and fracture risk. As eBMD is considered to be largely dependent on trabecular bone in the heel,[16] the reduced trabecular bone volume fraction in the vertebra of *Tac4*$^{-/-}$ mice might be unexpected. However, there could be underlying bone site-specific mechanisms or species differences.

Supplementary Note 8

Discussion on functional annotation and pathway analyses for the identified forearm fracture signals

Functional annotation revealed that nine of the identified forearm fracture signals in the present study have evidence that point to a single underlying gene. Among these, an amino acid altering variant (rs4988321, Val667Met) was identified in the established bone mass regulator LRP5[17], and strong MR support has been presented for low circulating RSPO3 being a causal risk factor for forearm fractures.[5] RSPO3 is a known regulator of bone metabolism both in humans and mice.[5,18] We also observed that the identified forearm fracture signal rs4671960 was a significant cis-eQTL for *SPTBN1* in osteoclasts. Genetic signals at the *SPTNB1* locus have previously been reported to associate with fracture risk at any bone site and eBMD.[11] [1] Furthermore, gene set enrichment analyses and pathway analyses identified known signaling pathways for bone metabolism associated with forearm fracture risk, including WNT signaling, Hedgehog signaling, and TGF-beta signaling.[17] Unexpectedly, a moderate enrichment for genes associated with systolic blood pressure was also observed, but the underlying biology influencing this relationship is unknown.

References (Supplementary Notes-only)

1. Morris, J.A., *et al.* An atlas of genetic influences on osteoporosis in humans and mice. *Nat Genet* **51**, 258-266 (2019).
2. Estrada, K., *et al.* Genome-wide meta-analysis identifies 56 bone mineral density loci and reveals 14 loci associated with risk of fracture. *Nat Genet* **44**, 491-501 (2012).
3. Medina-Gomez, C., *et al.* Life-Course Genome-wide Association Study Meta-analysis of Total Body BMD and Assessment of Age-Specific Effects. *Am J Hum Genet* **102**, 88-102 (2018).
4. Zheng, H.F., *et al.* Whole-genome sequencing identifies EN1 as a determinant of bone density and fracture. *Nature* **526**, 112-117 (2015).
5. Nilsson, K.H., *et al.* RSPO3 is important for trabecular bone and fracture risk in mice and humans. *Nat Commun* **12**, 4923 (2021).
6. Mullin, B.H., *et al.* Characterisation of genetic regulatory effects for osteoporosis risk variants in human osteoclasts. *Genome Biol* **21**, 80 (2020).
7. Kircher, M., *et al.* A general framework for estimating the relative pathogenicity of human genetic variants. *Nat Genet* **46**, 310-315 (2014).
8. Amendola, L.M., *et al.* Actionable exomic incidental findings in 6503 participants: challenges of variant classification. *Genome Res* **25**, 305-315 (2015).
9. Lamparter, D., Marbach, D., Rueedi, R., Kutalik, Z. & Bergmann, S. Fast and Rigorous Computation of Gene and Pathway Scores from SNP-Based Summary Statistics. *PLoS Comput Biol* **12**, e1004714 (2016).
10. Styrkarsdottir, U., *et al.* GWAS of bone size yields twelve loci that also affect height, BMD, osteoarthritis or fractures. *Nat Commun* **10**, 2054 (2019).
11. Trajanoska, K., *et al.* Assessment of the genetic and clinical determinants of fracture risk: genome wide association and mendelian randomisation study. *BMJ* **362**, k3225 (2018).
12. Nethander, M., *et al.* Assessment of the genetic and clinical determinants of hip fracture risk: Genome-wide association and Mendelian randomization study. *Cell Rep Med* **3**, 100776 (2022).
13. Andrew, T., Antioniades, L., Scurrah, K.J., Macgregor, A.J. & Spector, T.D. Risk of wrist fracture in women is heritable and is influenced by genes that are largely independent of those influencing BMD. *J Bone Miner Res* **20**, 67-74 (2005).
14. Ralston, S.H. & Uitterlinden, A.G. Genetics of osteoporosis. *Endocr Rev* **31**, 629-662 (2010).
15. Michaelsson, K., Melhus, H., Ferm, H., Ahlbom, A. & Pedersen, N.L. Genetic liability to fractures in the elderly. *Archives of internal medicine* **165**, 1825-1830 (2005).
16. Nethander, M., *et al.* BMD-Related Genetic Risk Scores Predict Site-Specific Fractures as Well as Trabecular and Cortical Bone Microstructure. *J Clin Endocrinol Metab* **105**(2020).
17. Lerner, U.H. & Ohlsson, C. The WNT system: background and its role in bone. *J Intern Med* **277**, 630-649 (2015).
18. Nilsson, K.H., *et al.* Estradiol and RSPO3 regulate vertebral trabecular bone mass independent of each other. *Am J Physiol Endocrinol Metab* **322**, E211-E218 (2022).

Supplementary Figures 1-8

A

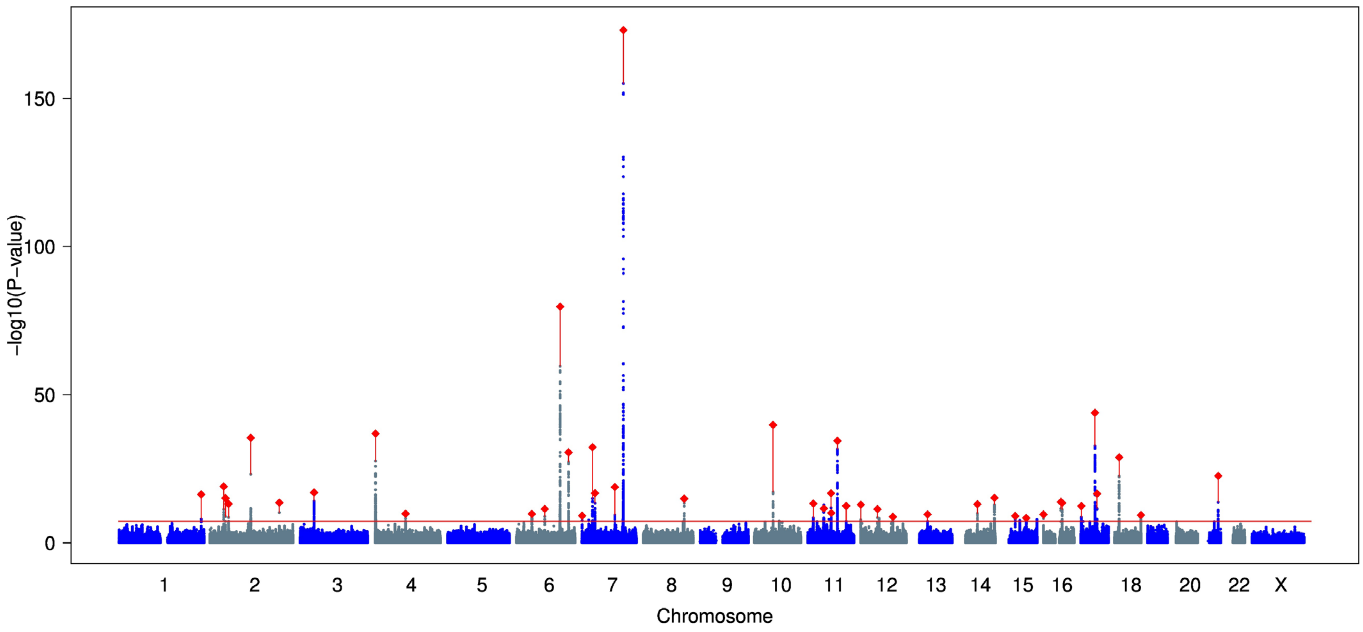

B

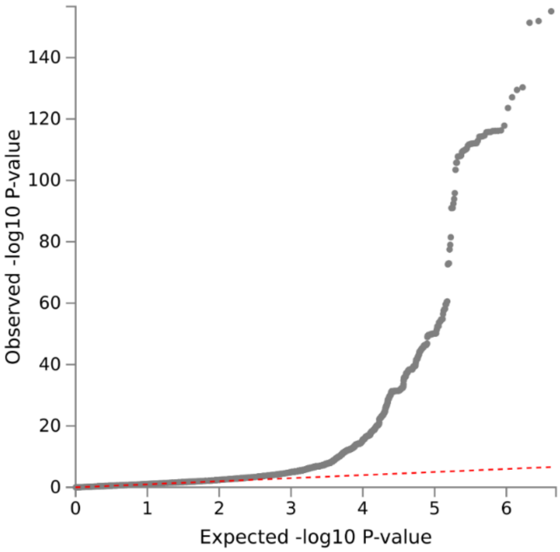

Supplementary Figure 1: Manhattan plot (A) and QQ-plot (B) of GWAS summary statistics: The grey and blue points in the Manhattan plot represents the negative 10-logarithm of the p-values from the discovery GWAS meta-analysis while the red dots represent the combined results after meta-analyses of the discovery analyses and the replication for the top signal in each of the 43 successfully replicated loci. The P-values are based on two-sided Z tests.

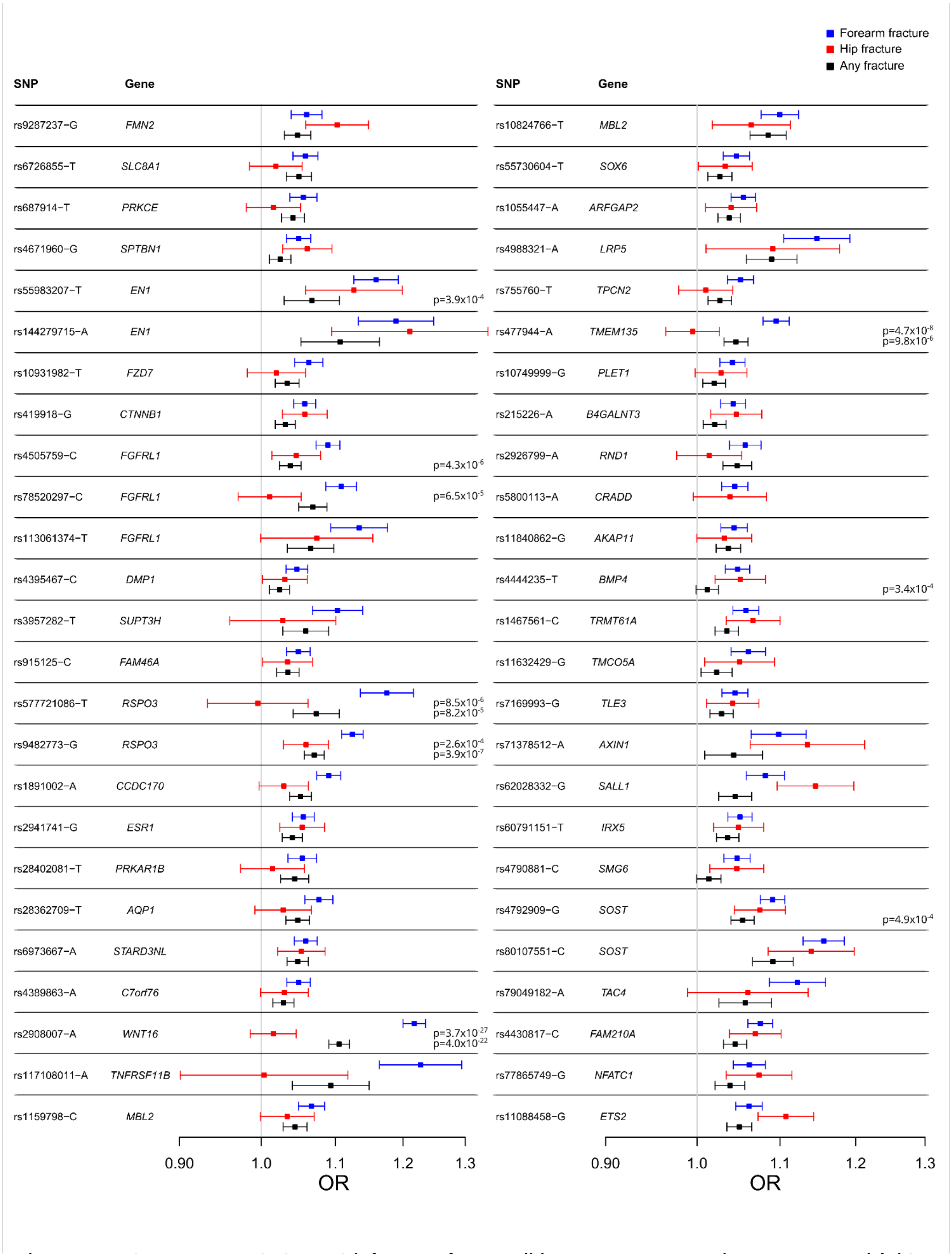

Supplementary Figure 2: Associations with forearm fracture (blue, 50,471 cases and 969,623 controls), hip fracture (red), and any fracture (black) risk for the identified top forearm fracture signals. Data are presented as odds ratios (OR) for fracture per effect allele, with 95% confidence intervals. OR for hip fractures are from Nethander *et al* 2022 (11,516 cases and 723,838 controls; PMID: 36260985), while OR for any fractures are from Morris *et al* 2020 (53,184 cases and 373,611 controls; PMID: 30598549). Significantly different associations for forearm fracture compared with the corresponding associations with hip fracture or any fracture are indicated with p-values. Two-sided z test was used to test differences and the significance limit was set to 0.0005 (Bonferroni adjustment considering 50 SNPs and 2 traits).

SNP	Gene	CADD	Coding	eQTL	P-MR
rs4988321	LRP5	**24.4**	■		
rs687914	PRKCE	**19.0**		■	
rs577721086	RSPO3	**17.6**		■	■
rs79049182	TAC4	**14.9**		Antisense	
rs10931982	FZD7	**13.5**			
rs62028332	SALL1	**13.0**			
rs9287237	GREM2	**7.1**		■	
rs28402081	PRKAR1B	**3.7**		■	
rs117108011	TNFRSF11B	**3.4**		■	
rs215226	B4GALNT3	**3.1**		■	
rs6726855	SLC8A1	**1.0**		■	

Supplementary Figure 3: Functional annotation. Variants (N = 9) with evidence pointing to a single effect-mediating gene in the form of protein coding (missense) variants (Coding), variants affecting mRNA expression (cis-eQTL, GTEx V8) or plasma protein MR evidence (P-MR), and additional variants (N= 2) with evidence of pathogenicity (CADD score ≥ 12.37 which is a suggested threshold for pathogenicity from a previous report, PMID 25637381) and not having multiple significant cis eQTL transcripts. In addition, the identified SNP in the *TAC4* locus was strongly associated with an eQTL for the TAC4 antisense RP11-304F15.3 ($p=2.6\times10^{-8}$ in blood, from weighted Z-score meta-analysis; https://www.eqtlgen.org/) located in the *TAC4* locus as indicated by “antisense” in the eQTL column in the figure.

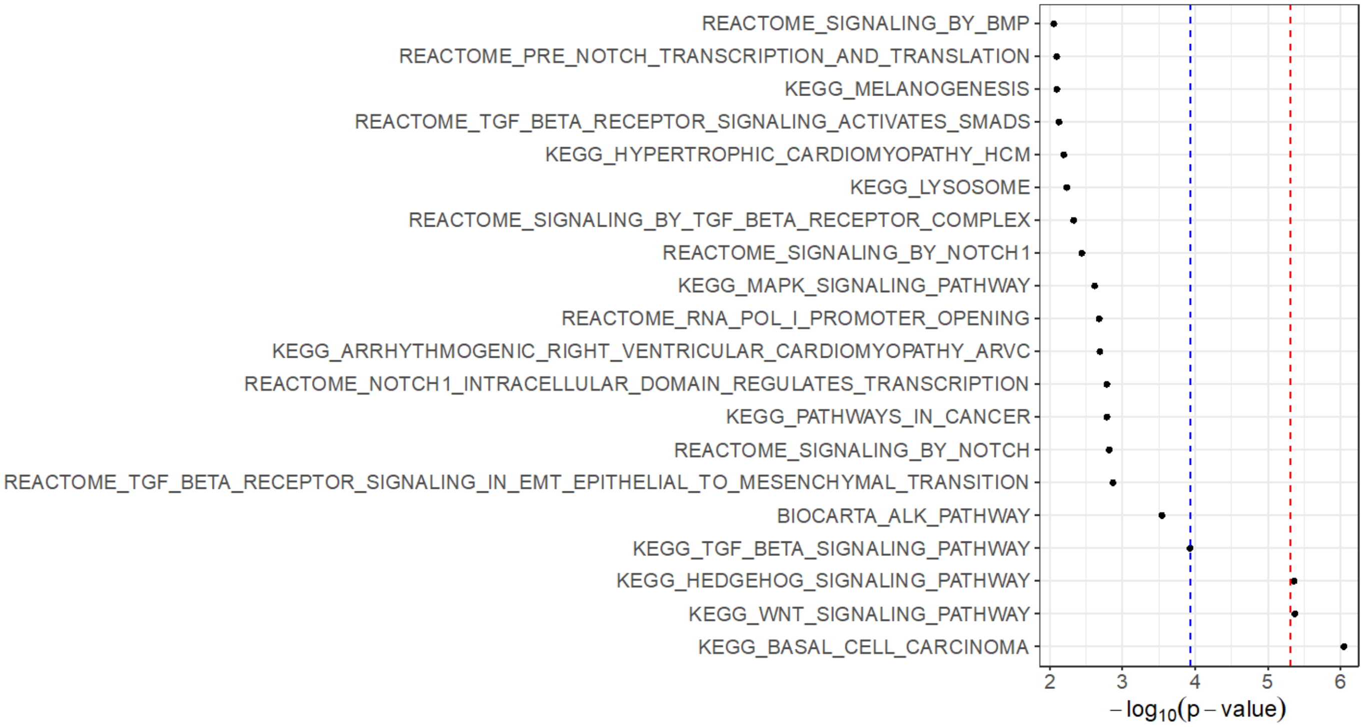

Supplementary Figure 4: PASCAL enrichment analyses. Biological pathway analysis using PASCAL gene set enrichment. The plot shows unadjusted negative log-10 p-values (one-sided) for the pathway scores from a chi^2 approximation, with the degree of freedom equal to the number of genes within each pathway. Only the top 20 gene sets are included in the plot. Red dashed line: Bonferroni-significance limit based on 1077 tested pathways. Blue dashed line: FDR-significant.

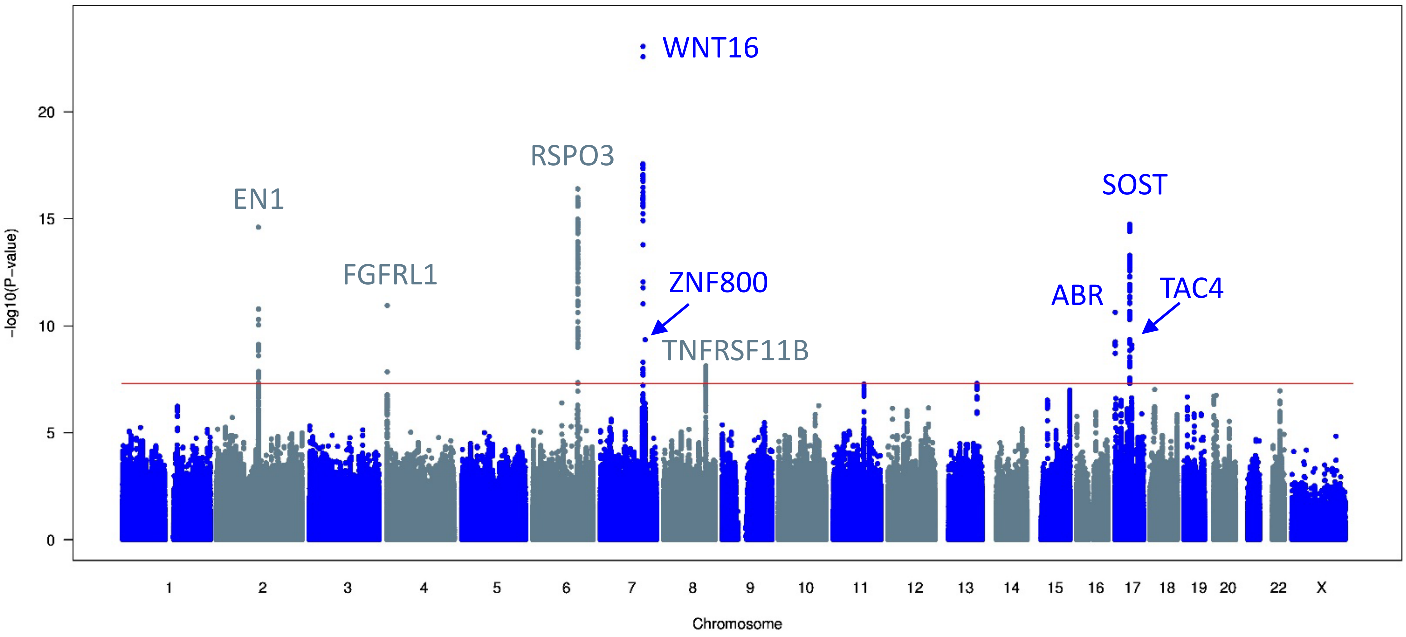

Supplementary Figure 5: Genome-wide significant signals for forearm fracture risk after removal of the genetic influence of eBMD on forearm fracture risk. We estimated the SNP effect on risk of fracture conditioned on eBMD using the mtCOJO tool. The Manhattan plot shows the results for risk of forearm fracture conditional on eBMD. Unadjusted p-values are from two-sided Z test. Significance level $p<5.0x10^{-8}$ (red line) was used.

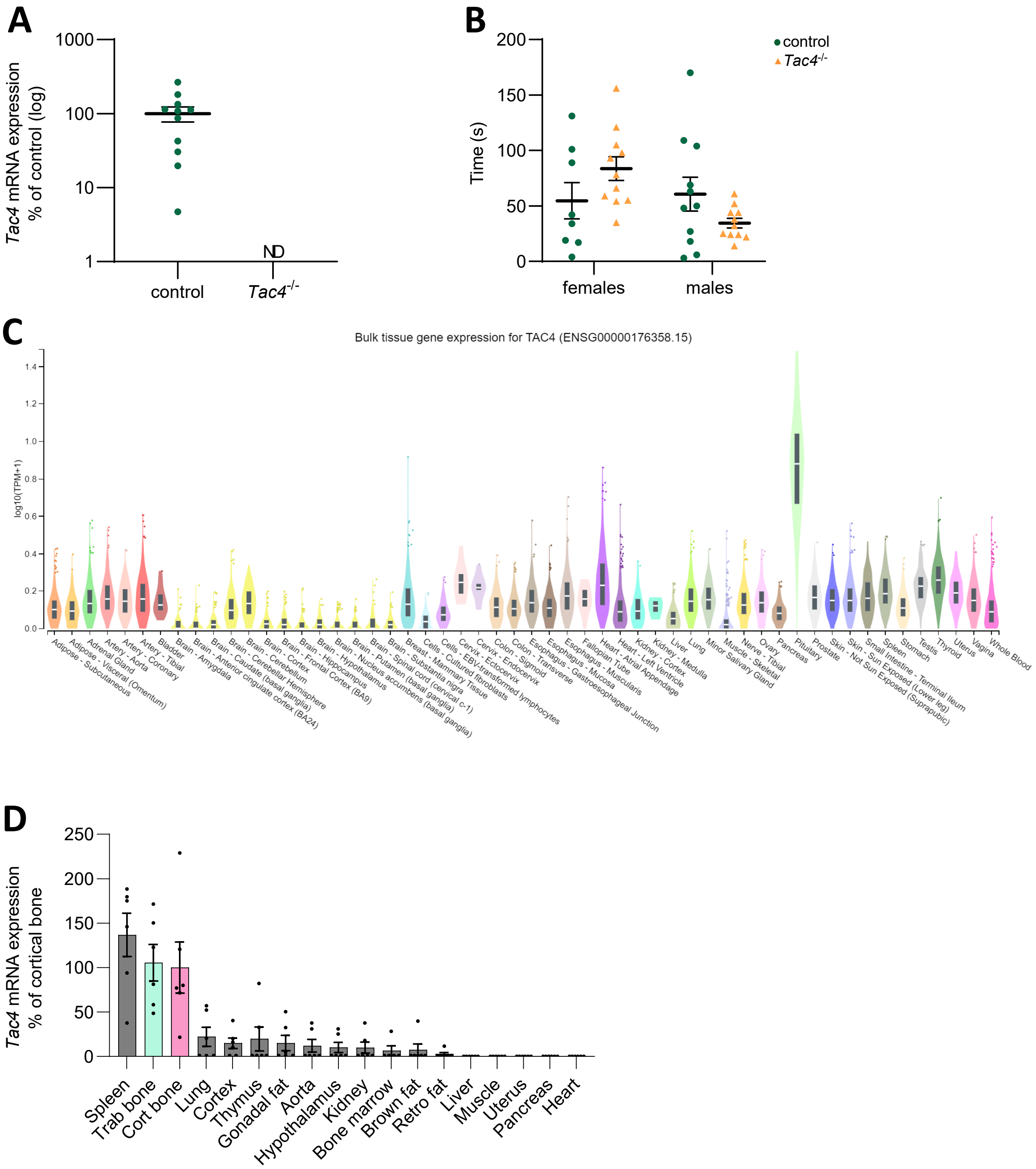

Supplementary Figure 6. *Tac4*$^{-/-}$ mice and expression of *TAC4* in different tissues.
A) mRNA expression analyses of *Tac4* in cortical bone of tibia in 12-month-old male control (n = 11) and *Tac4*$^{-/-}$ homozygote (n = 11) mice. Individual values are presented with the mean as horizontal lines and ± SEM as vertical lines. Values are log transformed. ND = not detectable. B) Motor coordination as examined by time spent on a rotating Rotarod wheel in 11-month-old *Tac4*$^{-/-}$ (females n = 11, males n = 11) mice compared to control (females n = 8, males n = 11) mice. Individual values are presented with the mean as horizontal lines and ± SEM as vertical lines. C) *TAC4* mRNA expression in multiple (n = 54) human tissues as available at the GTEx portal (https://gtexportal.org/home/). Bone tissue is not available at the GTEx portal. Box plots are shown as median and 25th and 75th percentiles. D) mRNA expression analyses of *Tac4* in 18 tissues of C57BL/6 female mice (n = 6). Individual values are presented with the mean as horizontal lines and ± SEM as vertical lines.

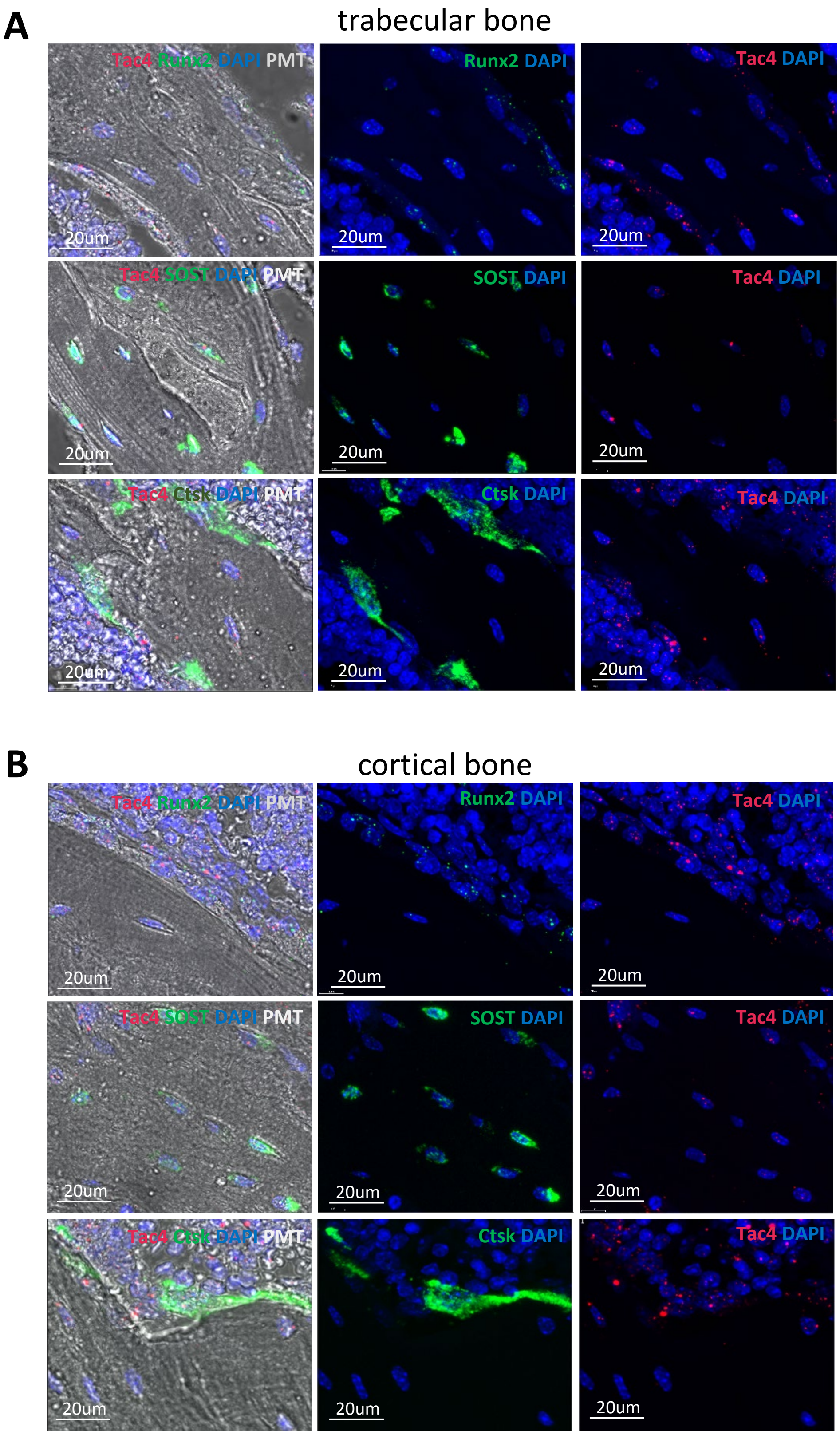

Supplementary Figure 7. Multiplex fluorescence in situ hybridization on mice tibiae.
Photo multipier tubes (PMT) demonstrate the structure of A) trabecular and B) cortical bone. Three confocal channels show DAPI nuclei staining (blue) and RNAscope signals for *Tac4* (red) and *Runx2*, *Sost* or *Ctsk* (green). RNAscope signals (displayed in Figure 3) are split in separate panels to help visualization of the target RNA. Representative maximum intensity projection images are shown of mice tibia. n = 3 biologically independent mice.

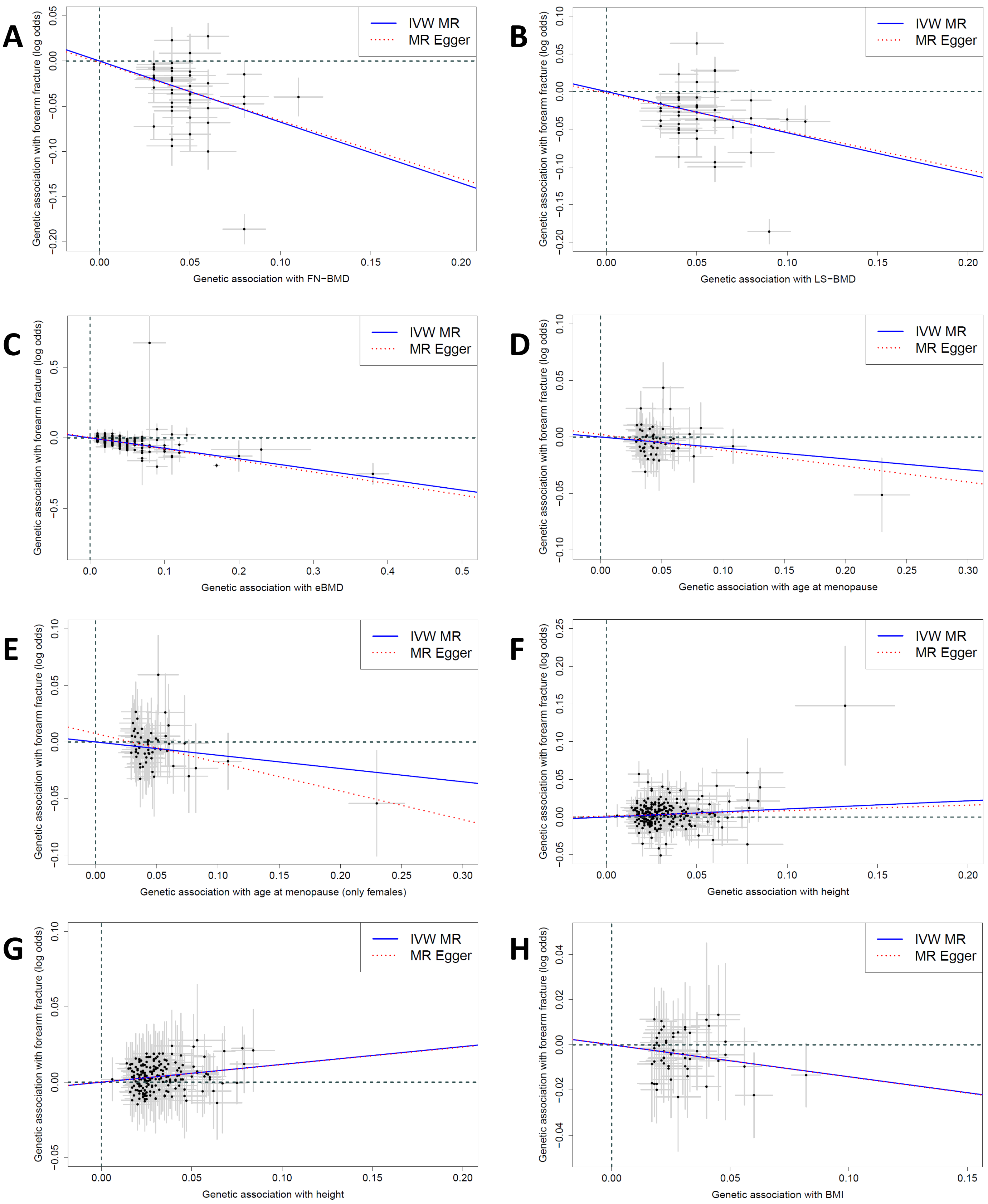

Supplementary Figure 8: Mendelian randomization plots. Effect estimates with 95% confidence intervals (CI) for the genetic association with different exposures (horizontal axis) are plotted against effect estimates with 95% CI for the genetic association with forearm fracture (vertical axis). Slopes were estimated using inverse variance-weighted (IVW) mendelian randomization (MR) and MR-Egger methods where the exposure variables were A) Femoral Neck Bone Mineral Density (FN-BMD), n instrument variables (IV) = 47; B) Lumbar Spine (LS) BMD, n IV = 45; C) estimated BMD (eBMD), n IV = 458; D) age at menopause, n IV = 54; E) age at menopause using only females, n IV = 54; F) height, n IV = 239; G) height with exclusion of outlier SNPs using MR-LASSO, n IV = 194 (See Supplementary Table 21); H) Body Mass Index (BMI), n IV = 55.
