# Supplementary material for: An atlas of genetic determinants of forearm fracture
Source: Nat Genet. 2023 Nov 2;55(11):1820–30. doi: 10.1038/s41588-023-01527-3 (PMC10632131; doi:10.1038/s41588-023-01527-3)
Supplement: Supplementary file 2 — Reporting Summary [file 41588_2023_1527_MOESM2_ESM.pdf]

nature portfolio

Corresponding author(s): Claes Ohlsson

Last updated by author(s): Aug 31, 2023

Reporting Summary

Nature Portfolio wishes to improve the reproducibility of the work that we publish. This form provides structure for consistency and transparency in reporting. For further information on Nature Portfolio policies, see our Editorial Policies and the Editorial Policy Checklist.

Statistics

For all statistical analyses, confirm that the following items are present in the figure legend, table legend, main text, or Methods section.

n/a	Confirmed	
☐	☒	The exact sample size (*n*) for each experimental group/condition, given as a discrete number and unit of measurement
☒	☐	A statement on whether measurements were taken from distinct samples or whether the same sample was measured repeatedly
☐	☒	The statistical test(s) used AND whether they are one- or two-sided<br>*Only common tests should be described solely by name; describe more complex techniques in the Methods section.*
☐	☒	A description of all covariates tested
☐	☒	A description of any assumptions or corrections, such as tests of normality and adjustment for multiple comparisons
☐	☒	A full description of the statistical parameters including central tendency (e.g. means) or other basic estimates (e.g. regression coefficient) AND variation (e.g. standard deviation) or associated estimates of uncertainty (e.g. confidence intervals)
☐	☒	For null hypothesis testing, the test statistic (e.g. *F*, *t*, *r*) with confidence intervals, effect sizes, degrees of freedom and *P* value noted<br>*Give P values as exact values whenever suitable.*
☒	☐	For Bayesian analysis, information on the choice of priors and Markov chain Monte Carlo settings
☒	☐	For hierarchical and complex designs, identification of the appropriate level for tests and full reporting of outcomes
☐	☒	Estimates of effect sizes (e.g. Cohen's *d*, Pearson's *r*), indicating how they were calculated

Our web collection on statistics for biologists contains articles on many of the points above.

Software and code

Policy information about availability of computer code

Data collection	NRecon 2.0.0.5..<br>CTan 1.12.2.1<br>Bluehill Universal software v4.25<br>StepOnePlus v2.3<br>Histolab software (Microvision, France)
Data analysis	We used the following publicly available software to analyze the data, described in detail in the manuscript.:<br>SAIGE v0.38 and PLINK v2.0 software were used to estimate the SNP associations (https://github.com/weizhouUMICH/SAIGE and https://www.cog-genomics.org/plink2/).<br>METAL (release 2011-03-25) software was used for fixed effect inverse-variance weighted meta-analysis (https://genome.sph.umich.edu/wiki/METAL).<br>GCTA v1.93 software package was used to identify conditionally independent SNPs (https://yanglab.westlake.edu.cn/software/gcta/#COJO).<br>FUMA v1.4.1 was used to characterize the genetic association signals of forearm fracture (https://fuma.ctglab.nl/).<br>ANNOVAR was used within FUMA, to categorize the functional sequences of the SNPs.<br>PASCAL enrichment analysis software was used to gain an overview of biological pathways (https://www2.unil.ch/cbg/index.php?title=Pascal).<br>LDSC v1.0.1 was used to estimate the genetic correlation between forearm fractures and other traits and diseases (https://github.com/bulik/ldsc).<br>The MR analyses were conducted using R v4.2.1 (https://cran.r-project.org/) and the R-packages MendelianRandomization v0.7.0 (https://cran.r-project.org/web/packages/MendelianRandomization/index.html) and MR-PRESSO v1 (https://github.com/rondolab/MR-PRESSO).

For manuscripts utilizing custom algorithms or software that are central to the research but not yet described in published literature, software must be made available to editors and reviewers. We strongly encourage code deposition in a community repository (e.g. GitHub). See the Nature Portfolio guidelines for submitting code & software for further information.

Data

Policy information about availability of data

All manuscripts must include a data availability statement. This statement should provide the following information, where applicable:

- Accession codes, unique identifiers, or web links for publicly available datasets
- A description of any restrictions on data availability
- For clinical datasets or third party data, please ensure that the statement adheres to our policy

Summary statistics from the GWAS meta-analysis are available at GWAS catalog (https://www.ebi.ac.uk/gwas/home). For cohort specific datasets each individual cohort has to be contacted as each country and cohort has different data access policies. Individual level Estonian Biobank data are available under restricted access administered by the Estonian Genome Center of the University of Tartu (EGCUT) in accordance with the regulations of the Estonian Human Genes Research Act. Access can be obtained by application at https://genomics.ut.ee/en. Individual level data from FinnGen participants can be accessed by approved researchers through the Fingenious portal (https://site.fingenious.fi/en/) hosted by the Finnish Biobank Cooperative FinBB (https://finbb.fi/en/). Access to UK biobank data can be obtained by application to UK biobank (https://www.ukbiobank.ac.uk/). Individual level data from DBDS cohort can be accessed by contacting the steering committee [info@dbds.dk]. Data access requires that projects and applicants obtain permission from the Regional Committees on Health Research Ethics and the Danish Data Protection Agency. Individual level data from HUNT can be accessed by, or in collaboration with a Norwegian principal investigator. Researchers can apply for HUNT data access from HUNT Research Centre (https://www.ntnu.edu/hunt) if they have obtained project approval from the Regional Committee for Medical and Health Research Ethics (REC). Information on the application and conditions for data access is available at https://www.ntnu.edu/hunt/data. For cohort specific data request of the remaining cohort used in the present forearm fracture study, contact Unnur Styrkarsdottir (unnur.Styrkarsdottir@decode.is) for the deCODE cohort, Ulrika Pettersson-Kymmer (ulrika.pettersson@umu.se) for the UFO cohort, and Sisse Rye Ostrowski (sisse.rye.ostrowski@regionh.dk) for the CHB-OF cohort.
All GWAS summary statistics used for risk factors in genetic correlations are available online: FN-BMD, LS-BMD, FA-BMD, eBMD and falls http://www.gefos.org/ ; age at menopause and age at menarche https://www.reprogen.org/data_download.html; relative age voice broke http://www.nealelab.is/uk-biobank; grip strength http://ldsc.broadinstitute.org/; vitamin D levels https://cnsgenomics.com/content/data; coronary artery disease http://www.cardiogramplusc4d.org/data-downloads/; rheumatoid arthritis http://plaza.umin.ac.jp/~yokada/datasource/software.htm; inflammatory bowel disease https://www.ebi.ac.uk/gwas/studies/GCST003043; type 2 diabetes https://diagram-consortium.org/downloads.html; smoking initiation and alcohol consumption (drinks per week) https://conservancy.umn.edu/handle/11299/201564; height and BMI https://portals.broadinstitute.org/collaboration/giant/index.php/GIANT_consortium_data_files.
All lookups were made in publicly available datasets and databases (GWAS catalog, CADD, GTEx V8 and Regulome DB via FUMA web application: https://fuma.ctglab.nl/; ChIP-Atlas: https://chip-atlas.org/peak_browser; previously published GWASs for FN-BMD, LS-BMD, eBMD and any fracture http://www.gefos.org/ ; and previously published GWAS for hip fracture https://www.ebi.ac.uk/gwas/publications/36260985).

Field-specific reporting

Please select the one below that is the best fit for your research. If you are not sure, read the appropriate sections before making your selection.

☒ Life sciences ☐ Behavioural & social sciences ☐ Ecological, evolutionary & environmental sciences

For a reference copy of the document with all sections, see nature.com/documents/nr-reporting-summary-flat.pdf

Life sciences study design

All studies must disclose on these points even when the disclosure is negative.

Sample size	For the discovery meta-analysis, we included subjects from five Northern European biobanks with forearm fracture data and genotype data available. In total, 50,471 forearm fracture cases and 969,623 controls were included in the discovery meta-analysis. Replication analyses of GWS hits were performed in three other large biobank samples including 49,555 forearm fracture cases and 620,360 controls. In total, 100,026 forearm fracture cases and 1,589,983 controls were included in the present discovery and replication analyses. Sample sizes for both discovery and replication were chosen on the basis of all data available at the time for analysis.
Data exclusions	To reduce potential bias due to population stratification, we restricted the analyses to participants of European descent in four of the biobanks used in the discovery meta-analysis.
Replication	Replication analyses of GWS hits were performed in three other large biobank samples including 49,555 forearm fracture cases and 620,360 controls. 59 SNPs were tested in the replication part and 50 SNPs were successfully replicated ($p<0.05$ with the effect estimate in the same direction as in the discovery analysis, see supplementary table 5).
Randomization	Not applicable (GWAS study, not a randomized trial)
Blinding	Not applicable (GWAS study, not a randomized trial)

Reporting for specific materials, systems and methods

We require information from authors about some types of materials, experimental systems and methods used in many studies. Here, indicate whether each material, system or method listed is relevant to your study. If you are not sure if a list item applies to your research, read the appropriate section before selecting a response.

Materials & experimental systems

n/a	Involved in the study
☒	☐ Antibodies
☒	☐ Eukaryotic cell lines
☒	☐ Palaeontology and archaeology
☐	☒ Animals and other organisms
☐	☒ Human research participants
☒	☐ Clinical data
☒	☐ Dual use research of concern

Methods

n/a	Involved in the study
☒	☐ ChIP-seq
☒	☐ Flow cytometry
☒	☐ MRI-based neuroimaging

Animals and other organisms

Policy information about studies involving animals; ARRIVE guidelines recommended for reporting animal research

Laboratory animals	Female and male Tac4-deficient (Tac4-/-) and wild type C57Bl/6 mice were used in the study. N = 9-11. Age of termination = 12-month-old. Animals were kept under a standard 12-h light/dark cycle in 50-60% humidity at a temperature of 24 ± 2 °C.
Wild animals	No wild animals were used in this study.
Field-collected samples	No field-collected samples were used in this study.
Ethics oversight	All procedures were performed according to the European legislation (Directive 2010/63/EU) and Hungarian Government regulation (40/2013., II. 14.) and were approved by the National Ethics Committee on Animal Research of Hungary (license No.: BA/73/00657-3/2022).

Note that full information on the approval of the study protocol must also be provided in the manuscript.

Human research participants

Policy information about studies involving human research participants

Population characteristics	See detailed description in supplemental tables 1-4 for the eight included biobanks
Recruitment	See detailed description in supplemental tables 1-4 for the eight included biobanks
Ethics oversight	The UK Biobank has ethical approval from the Northwest Multicentre Research Ethics Committee, and informed consent was obtained from all participants. The present research was approved by the UK Biobank Research and Access Committee (application number 51784).<br><br>Patients and control subjects in FinnGen provided informed consent for biobank research, based on the Finnish Biobank Act. Alternatively, separate research cohorts, collected prior the Finnish Biobank Act came into effect (in September 2013) and start of FinnGen (August 2017), were collected based on study-specific consents and later transferred to the Finnish biobanks after approval by Fimea (Finnish Medicines Agency), the National Supervisory Authority for Welfare and Health. Recruitment protocols followed the biobank protocols approved by Fimea. The Coordinating Ethics Committee of the Hospital District of Helsinki and Uusimaa (HUS) statement number for the FinnGen study is Nr HUS/990/2017. The FinnGen study is approved by Finnish Institute for Health and Welfare (permit numbers: THL/2031/6.02.00/2017, THL/1101/5.05.00/2017, THL/341/6.02.00/2018, THL/2222/6.02.00/2018, THL/283/6.02.00/2019, THL/1721/5.05.00/2019 and THL/1524/5.05.00/2020), Digital and population data service agency (permit numbers: VRK43431/2017-3, VRK/6909/2018-3, VRK/4415/2019-3), the Social Insurance Institution (permit numbers: KELA 58/522/2017, KELA 131/522/2018, KELA 70/522/2019, KELA 98/522/2019, KELA 134/522/2019, KELA 138/522/2019, KELA 2/522/2020, KELA 16/522/2020), Findata permit numbers THL/2364/14.02/2020, THL/4055/14.06.00/2020,,THL/3433/14.06.00/2020, THL/4432/14.06/2020, THL/5189/14.06/2020, THL/5894/14.06.00/2020, THL/6619/14.06.00/2020, THL/209/14.06.00/2021, THL/688/14.06.00/2021, THL/1284/14.06.00/2021, THL/1965/14.06.00/2021, THL/5546/14.02.00/2020, THL/2658/14.06.00/2021, THL/4235/14.06.00/2021 and Statistics Finland (permit numbers: TK-53-1041-17 and TK/143/07.03.00/2020 (earlier TK-53-90-20) TK/1735/07.03.00/2021). The Biobank Access Decisions for FinnGen samples and data utilized in FinnGen Data Freeze 8 include: THL Biobank BB2017_55, BB2017_111, BB2018_19, BB_2018_34, BB_2018_67, BB2018_71, BB2019_7, BB2019_8, BB2019_26, BB2020_1, Finnish Red Cross Blood Service Biobank 7.12.2017, Helsinki Biobank HUS/359/2017, Auria Biobank AB17-5154 and amendment #1 (August 17 2020), AB20-5926 and amendment #1 (April 23 2020), Biobank Borealis of Northern Finland_2017_1013, Biobank of Eastern Finland 1186/2018 and amendment 22 § /2020, Finnish Clinical Biobank Tampere MH0004 and amendments (21.02.2020 & 06.10.2020), Central Finland Biobank 1-2017, and Terveystalo Biobank STB 2018001.<br><br>Estonian Biobank has ethical approval from the Estonian Commitee on Bioethics and Human Research at the Ministry of

Social Affairs (No 1.1-12/624). Written informed consent for participation was obtained from all study subjects.

Participation in the HUNT Study is based on informed consent and the study has been approved by the Regional Ethics Committee for Medical Research in Norway (REK 4.2006.250; Sak 142-99; REK 2014/144).

The UFO study was approved by the local research ethics committee at Umeå University (Umu dnr 03-426; EPN 2012-254-32M; 2011/32-32M; 2011-251-32M). Written consent was obtained from all participants.

The deCODE study were approved by the National Bioethics Committee (NBC). The data in this study was approved by the NBC (VSN-15-198) following review by the Icelandic Data Protection Authority. All personal identifiers of the participants' data were encrypted in accordance with the regulations of the Icelandic Data Protection Authority. Participants donated blood or buccal samples after signing a broad informed consent.

The Copenhagen Hospital Biobank (CHB) is a research biobank in the Danish Capital Region hospitals. Under the "Genetics of osteoporosis and fractures" protocol (CHB-OF), approved by the Danish Data Protection Agency (P-2019-542) and the National Committee on Health Research Ethics (NVK-1903714), forearm fracture cases and controls were included.

The Danish Blood Donor Study (DBDS) Genomic Cohort is a study approved by the The Danish Data Protection Agency (P-2019-99) and the National Committee on Health Research Ethics (NVK-1700407) approved the studies under which genetic data on DBDS participants were obtained. The DBDS data requested for this study was approved by the DBDS steering committee.

Note that full information on the approval of the study protocol must also be provided in the manuscript.
